# Regulatory effects of Sini-San on bile acid homeostasis in the enterohepatic circulation of mice with liver fibrosis

**DOI:** 10.1186/s13020-025-01252-5

**Published:** 2025-11-11

**Authors:** Fangsi Zhu, Yijie Ding, Luyun Chen, Rou Fang, Chengfeng Huang, En Liu, Yingrui Wang, Yong Su, Chaoliang Ge

**Affiliations:** 1 Department of Pharmacy, The First Affiliated Hospital of Anhui Medical University, Hefei, 230032 Anhui China; 2 School of Pharmacy, Anhui Medical University, Hefei, 230032 Anhui China

**Keywords:** Sini-San, Bile acids homeostasis, Intestinal flora, FXR, Liver fibrosis

## Abstract

**Background:**

Sini-San (SNS), a classical traditional Chinese medicinal formula, has demonstrated promising potential in mitigating the progression of liver fibrosis (LF). Increasing evidence highlights that disruption of bile acids (BAs) homeostasis is critically involved in the pathogenesis and progression of LF, suggesting that targeting BAs metabolism could represent a therapeutic strategy. This study aimed to explore whether the protective effects of SNS against LF are mediated through modulation of BAs metabolism and associated regulatory pathways.

**Methods:**

The chemical constituents of SNS were characterized using high-performance liquid chromatography (HPLC). LF models were established in mice through intraperitoneal injection of carbon tetrachloride (CCl_4_) or feeding a high-fat, high-sugar (HFHS) diet. SNS was administered orally. Serum alanine aminotransferase (ALT), aspartate aminotransferase (AST) and hydroxyproline (HYP) levels were measured, and liver histopathology was evaluated by hematoxylin–eosin (HE), Masson and TUNEL staining. The expression of fibrosis- and apoptosis-associated markers (Collagen-1, α-SMA, Bcl-2, Bax, and Caspase-3) was assessed by RT-qPCR and Western blotting. Serum BAs profiles were analyzed using LC–MS/MS, and molecules involved in BA metabolism (Fxr, Cyp7a1, Cyp27a1, Bsep, Ntcp, Asbt and OATP) were examined. Gut microbiota composition was analyzed through 16S rRNA gene sequencing. To investigate the mechanisms by which SNS regulates BAs homeostasis, additional experiments were conducted under choline chelation, pseudo-sterile conditions, and in *fxr*^*−/−*^ mice.

**Results:**

In LF mice induced by CCl_4_ or HFHS diet, significant alterations were observed in BAs levels and composition. The expression of BAs-synthesizing enzymes (CYP7A1, CYP27A1), BAs transporters (*Bsep, Ntcp, Asbt* and *Oatp*), and the feedback regulatory receptor FXR was markedly dysregulated. Meanwhile, gut microbiota abundance and composition were also significantly disrupted, indicating a disturbance of BAs homeostasis. SNS treatment effectively alleviated liver injury and fibrosis, corrected BAs imbalance, regulated the expression of BAs-related genes, and restored microbial diversity. However, the antifibrotic effects of SNS were reversed by choline chelation, antibiotic treatment, and *fxr* knockout.

**Conclusions:**

SNS may exert anti-hepatic fibrosis effects by modulating BAs metabolism and gut-liver axis pathways, ultimately restoring BAs homeostasis. These findings provide new insights into the therapeutic mechanisms of SNS and suggest its potential as a multitargeted strategy for LF treatment.

**Supplementary Information:**

The online version contains supplementary material available at 10.1186/s13020-025-01252-5.

## Introduction

The high prevalence and mortality rates of cirrhosis and hepatocellular carcinoma pose a major global health challenge [1]. As a critical and reversible stage in the progression of these diseases, liver fibrosis (LF) lacks effective pharmacotherapies, highlighting an urgent need for therapeutic interventions [2].

Bile acids (BAs) are crucial endogenous secretory products of the liver. Beyond their role in fat digestion, they serve as critical signaling molecules essential for maintaining the normal function of the liver, intestine, and other organs [3]. Dysregulated BAs, a hallmark of liver diseases such as non-alcoholic fatty liver disease (NAFLD) and hepatocellular carcinoma (HCC), are critical contributors to LF. Persistent external stimuli or injuries can disrupt BAs composition and concentrations, promoting LF progression [4].

BAs homeostasis is maintained through three fundamental physiological processes: biosynthesis, metabolism, and enterohepatic circulation. The composition and abundance of intestinal microbiota, along with the expression of BAs-specific receptors and transporter proteins, play a crucial role in shaping BAs pool composition and maintaining its stability. These factors collectively regulate BAs homeostasis, which in turn influences LF progression [5]. For instance, increased intestinal permeability permit intestinal microbes and their metabolites, including BAs, to enter systemic circulation, activating inflammatory pathways and exacerbating LF [6]. Conversely, the farnesoid X receptor (FXR), a primary sensor of BAs concentrations, mitigates LF by suppressing BAs synthesis and promoting excretion [7].

Sini-San (SNS), a traditional Chinese herbal formula documented in the *Treatise on Febrile Diseases*, is used to treat chronic hepatitis, fatty liver, and hepatocellular carcinoma [8–10]. Our previous study demonstrated that SNS alleviated CCl_4_-induced LF and restored hepatic FXR protein expression [11]. Previous study has also shown that SNS significantly down-regulates the levels of glycochenodeoxycholic acid (GCDCA) in liver-injured mice [12]. Several studies have shown that the active constituents of SNS regulate BAs metabolism. For example, saikosaponins activate the FXR and BAs transporters to regulate BAs excretion in the liver and ileum of mice [13]. Additionally, SNS has been reported to alleviate colonic injury by enhancing intestinal barrier function, modulating gut microbiota composition, and regulating cholesterol metabolism [14, 15]. These data suggest that SNS may be involved in the regulation of BAs metabolism.

## Materials and methods

### Preparation and component analysis of SNS

SNS consists of *Citrus* × *aurantium* f. aurantium [Rutaceae, fructus immaturus] (Anhui Puren, 2108231), *Glycyrrhiza uralensis* Fisch. [Leguminosae, Glycyrrhiza uralensis Fisch.ex DC, radix et rhizoma] (Anhui Puren, 2107191), *Paeonia lactiflora* Pall. [Paeoniaceae, radix] (Anhui Puren, 2012091) and *Bupleurum falcatum* L. [Apiaceae, radix] (Anhui Puren, 2104123) in a ratio of 1:1:1:1. The preparation involved adding ten volumes of purified water to the crude materials for an initial 1.5-h decoction, after which the extract was collected. The remaining residue underwent a second extraction with eight volumes of water, and the liquid was again harvested. A third decoction was carried out by adding six volumes of water and boiling for 0.5 h. All three extracts were subsequently combined, filtered through a lint-free cloth to eliminate insoluble residues, and concentrated to achieve a final concentration of 0.31 g/mL [11].

### Component analysis of SNS

The four herbal components of SNS were combined in equal ratios (1:1:1:1) to prepare a total of 100 g of crude material. The aqueous extract was obtained by soaking this mixture in distilled water, followed by extraction and subsequent concentration to a final volume of 1000 mL. Quantitative analysis was conducted using high-performance liquid chromatography (HPLC) equipped with an Agilent ZORBAX SB-C_18_ column (4.6 × 150 mm, 5 μm). The mobile phase included acetonitrile (A) and 0.4% aqueous phosphoric acid (B). UV detection was performed at 210, 240, 254, and 360 nm with a flow rate of 1.0 mL/min. The column temperature was set at 30 °C. Gradient elution was applied as follows: 0–10 min, 15–17% A; 10–20 min, 17–25% A; 20–26 min, 25–35% A; 26–35 min, 35% A; 35–45 min, 35–50% A; and 45–63 min, 80% A.

### Animal treatment

Six-week-old male C57BL/6 J mice (SPF grade) were obtained from Hefei Qingyuan Biotechnology Co., Ltd. (No. 2107262457842654) and housed at the Anhui Academy of Medical Sciences (No. SYXK (Anhui) 2019–008). Animals were kept under controlled environmental conditions: a temperature of 22 ± 2 °C, relative humidity of 40–70%, and a 12-h light/dark cycle. Food and water were provided ad libitum. All animal procedures were reviewed and approved by the Ethics Committee of Anhui Medical University (Approval No. LLSC20210242).

### Establishment of LF mouse model

In this experiment, two models of LF induction in mice were used: CCl_4_ and high-fat, high-sugar (HFHS). Following a 1-week acclimation period, the animals were randomly assigned to three experimental groups. For the CCl_4_-induced fibrosis model, mice in the model (CCl_4_) and treatment (CCl_4_ + SNS) groups received intraperitoneal injections of CCl_4_ (0.5 mL/kg; Macklin, C805329) diluted in olive oil (Yuanye, S30503) three times weekly over 6 weeks. During the final 4 weeks, the CCl_4_ + SNS group was administered SNS via oral gavage at a dose of 6.2 g/kg, based on the optimal therapeutic dose identified in our previous research [11]. For HFHS-induced LF, HFHS chow (customized by Jiangsu Synergistic Pharmaceutical and Bioengineering Co., Ltd.), which contained 40% fat and 43% carbohydrates, and high-sugar drinking water supplemented with 23.1 g/L fructose (Biosharp, BS913) and 18.9 g/L sucrose (Biosharp, BS085), was provided to both the model group (HFHS) and the drug treatment group (HFHS + SNS) for a total of 24 weeks. During the last 4 weeks of the modeling period, mice in the HFHS + SNS group were gavaged daily with SNS (6.2 g/kg).

### Establishment of LF model in mice after BAs chelation

In this experiment, we established a mouse model of LF using cholestyramine (Sigma-Aldrich, C4650) as a BAs chelator. For the CCl_4_-induced LF model, the mice were divided into the following six groups: normal (Control), model (CCl_4_), administration (CCl_4_ + SNS), cholestyramine (Cholestyramine), cholestyramine model (Cholestyramine + CCl_4_), and cholestyramine administration (Cholestyramine + CCl_4_ + SNS). For the HFHS-induced LF model, the groups included: normal (Control), model (HFHS), administration (HFHS + SNS), cholestyramine (Cholestyramine), cholestyramine model (Cholestyramine + HFHS), and cholestyramine administration (Cholestyramine + HFHS + SNS). The cholestyramine groups were fed a special diet (customized by Jiangsu Synergistic Pharmaceutical and Bioengineering Co., Ltd.) supplemented with 2% cholestyramine during the final 4 weeks of the modeling period. The induction methods for the two LF models were consistent with those described earlier.

### Establishment of LF model in pseudo-sterile mice

In this experiment, CCl_4_ and HFHS-induced LF models were established in pseudo-sterile mice. These pseudo-sterile mice were housed in an SPF-grade animal facility. Taking into account the differences in modeling duration between the CCl_4_ and HFHS induced liver fibrosis models and the palatability issues associated with antibiotic administration, different antibiotic intervention approaches were applied in each model, as described below. For the CCl_4_-induced LF model in pseudo-sterile mice, the animals were assigned to six groups as follows: normal (Control), model (CCl_4_), drug administration (CCl_4_ + SNS), pseudo-sterile (Antibiotic), pseudo-sterile model (Antibiotic + CCl_4_), and pseudo-sterile drug administration (Antibiotic + CCl_4_ + SNS). In the pseudo-sterile groups, antibiotics were added to the drinking water of the mice based on the normal CCl_4_-induced LF model. The antibiotics used included: 0.5 g/L vancomycin (Aladdin, V10549), 1 g/L neomycin sulfate (Aladdin, N109017), 1 g/L ampicillin (Aladdin, A105483), and 1 g/L metronidazole (Aladdin, M109874) administered for 6 weeks. For the HFHS-induced LF model in pseudo-sterile mice, the animals were assigned to six groups as follows: normal (Control), model (HFHS), drug administration (HFHS + SNS), pseudo-sterile (Antibiotic), pseudo-sterile model (Antibiotic + HFHS), and pseudo-sterile drug administration (Antibiotic + HFHS + SNS). In the pseudo-sterile groups, antibiotics (10 mg/mL vancomycin, 20 mg/mL neomycin sulfate, 20 mg/mL ampicillin, and 20 mg/mL metronidazole) were administered by daily gavage, starting 10 days prior to SNS gavage. The dosage was 0.2 mL per mouse per day, and this regimen continued until the end of the modeling period [16]. The induction methods for the two LF models were consistent with those described earlier.

### Establishment of a CCl_4_-induced LF model in fxr^−/−^ mice

The *fxr*^*−/−*^ mice were kindly provided by the group of Prof. Hao Haiping from China Pharmaceutical University. The mice were divided into six groups: wild-type (WT), wild-type model (WT CCl_4_), wild-type administration (WT CCl_4_ + SNS), knockout mice (*fxr*^*−/−*^), knockout model (*fxr*^*−/−*^ CCl_4_), and knockout administration (*fxr*^*−/−*^ CCl_4_ + SNS). The method for inducing CCl_4_-induced LF in the knockout mice was consistent with that used for the wild-type mice.

### 16S rRNA amplicon sequencing

Genomic DNA was extracted from the samples using the MagPure Soil DNA LQ Kit (Magan) following the manufacturer’s protocol. DNA concentration and quality were evaluated using a NanoDrop 2000 spectrophotometer (Thermo Fisher Scientific, USA) and agarose gel electrophoresis. The isolated DNA served as the template for PCR amplification of the bacterial 16S rRNA gene, employing barcoded primers and Takara Ex Taq polymerase (Takara). Amplicon integrity was verified by gel electrophoresis. PCR products were purified using AMPure XP magnetic beads (Agencourt), followed by a second round of amplification. After an additional purification step with AMPure XP beads, the final amplicons were quantified using the Qubit dsDNA Assay Kit (Thermo Fisher Scientific, USA). DNA concentrations were then normalized and sequenced on the Illumina NovaSeq 6000 platform with 250 bp paired-end reads (Illumina Inc., San Diego, CA), performed by OE Biotech Co., Ltd. (Shanghai, China) [17].

### Serum BAs analysis

BAs profiles were analyzed same as described previously [18]. BAs were quantified following established protocols. For sample preparation, 100 μL of serum was diluted with 500 μL of 0.01% formic acid, using dehydrocholic acid as the internal standard. The diluted sample was loaded onto an Oasis-HLB column, washed with 1 mL of water, and eluted with 1.5 mL of methanol. After evaporation, the residue was redissolved in 100 μL of methanol. Analysis was performed using an HPLC system (Shimadzu, Kyoto, Japan) coupled with an AB Sciex Triple TOF 5600 (HPLC-QTOF, AB Sciex, Foster City, CA, USA) and a ZORBAX Eclipse Plus C18 column. A 5 μL injection volume was used [19].

### Biochemical analysis

Experiments were conducted using a fully automated biochemical analyzer to measure serum alanine aminotransferase (ALT) and aspartate aminotransferase (AST) levels, which serve as indicators of liver injury in mice. Hydroxyproline (HYP) and total bile acid (TBA) in serum was measured using a commercial assay kit (Nanjing Jiancheng, A030-2-1 and E003-2-1).

### Histopathology

Liver tissues were collected from mice, fixed, and embedded in paraffin. The paraffin blocks were sectioned into 5 μm slices. Hematoxylin and eosin (HE) staining was performed to assess tissue and cellular morphology, Masson’s staining was used to evaluate fibrosis deposition, and TUNEL staining was conducted to detect hepatocyte apoptosis.

### Western blot analysis

The liver and intestinal tissue from the mice was homogenized and lysed in RIPA buffer (Beyotime, P0013C) containing a protease inhibitor (Beyotime, ST506). A total of 20–30 μg of protein was subjected to SDS-PAGE. The proteins were transferred, blocked, and then incubated with the primary antibody at 4 °C overnight. On the subsequent day, the membrane was incubated with the corresponding secondary antibody for 1 h at room temperature. On the second day, the membrane was again incubated with the corresponding secondary antibody for 1 h at room temperature. Following a TBST wash, the blot was visualized using a luminescence instrument (ChemiDoc MP, Bio-Rad) and the grey scale values of the protein bands were quantified using ImageJ. The antibody models, manufacturers, and dilution ratios are listed in the Table S1.

### Quantitative PCR analysis

Total RNA was isolated from mouse liver tissue using the Trizol reagent (Invitrogen, 15596-026). RNA concentration and purity were measured with a NanoDrop micro-UV spectrophotometer (Thermo Scientific, USA). Complementary DNA (cDNA) was synthesized using the Evo M-MLV RT Pre-mix (Accurate Biology, AG11706). Quantitative real-time PCR (qRT-PCR) was conducted on a LightCycler 480 instrument (Roche). The relative mRNA expression levels of *Collagen-I*, *α-SMA*, *Fxr, Cyp7a1, Cyp27a1, Bsep, Ntcp, Asbt* and *Oatp* were calculated using the 2^^–ΔΔCt^ method. Primer sequences are provided in Table S2.

### Statistical analysis

All experiments were conducted at least three times and analyzed statistically using GraphPad Prism 8.0 or IBM SPSS Statistics 26. Data are presented as mean ± standard error of the mean (SEM). One-way ANOVA with Tukey’s post-hoc test was applied for normally distributed data, while the Kruskal–Wallis test was used for non-normally distributed data. P < 0.05 indicates statistically significant differences.

## Results

### SNS ingredient analysis

SNS consists of *Citrus* × *aurantium* f. aurantium [Rutaceae, fructus immaturus] (Anhui Puren, 2108231), *Glycyrrhiza uralensis* Fisch. [Leguminosae, Glycyrrhiza uralensis Fisch.ex DC, radix et rhizoma] (Anhui Puren, 2107191), *Paeonia lactiflora* Pall. [Paeoniaceae, radix] (Anhui Puren, 2012091) and *Bupleurum falcatum* L. [Apiaceae, radix] (Anhui Puren, 2104123) in a ratio of 1:1:1:1. Based on the specifications outlined in the Pharmacopoeia of the People’s Republic of China (2020) and relevant literature [20, 21], 10 constituents, including albiflorin, paeoniflorin and hesperidin, were selected as reference standards. These components were successfully identified in the aqueous extract of SNS (Fig. S1).

### SNS attenuates CCl_4_ or HFHS-induced liver injury and fibrosis in mice

**Fig. 1:**
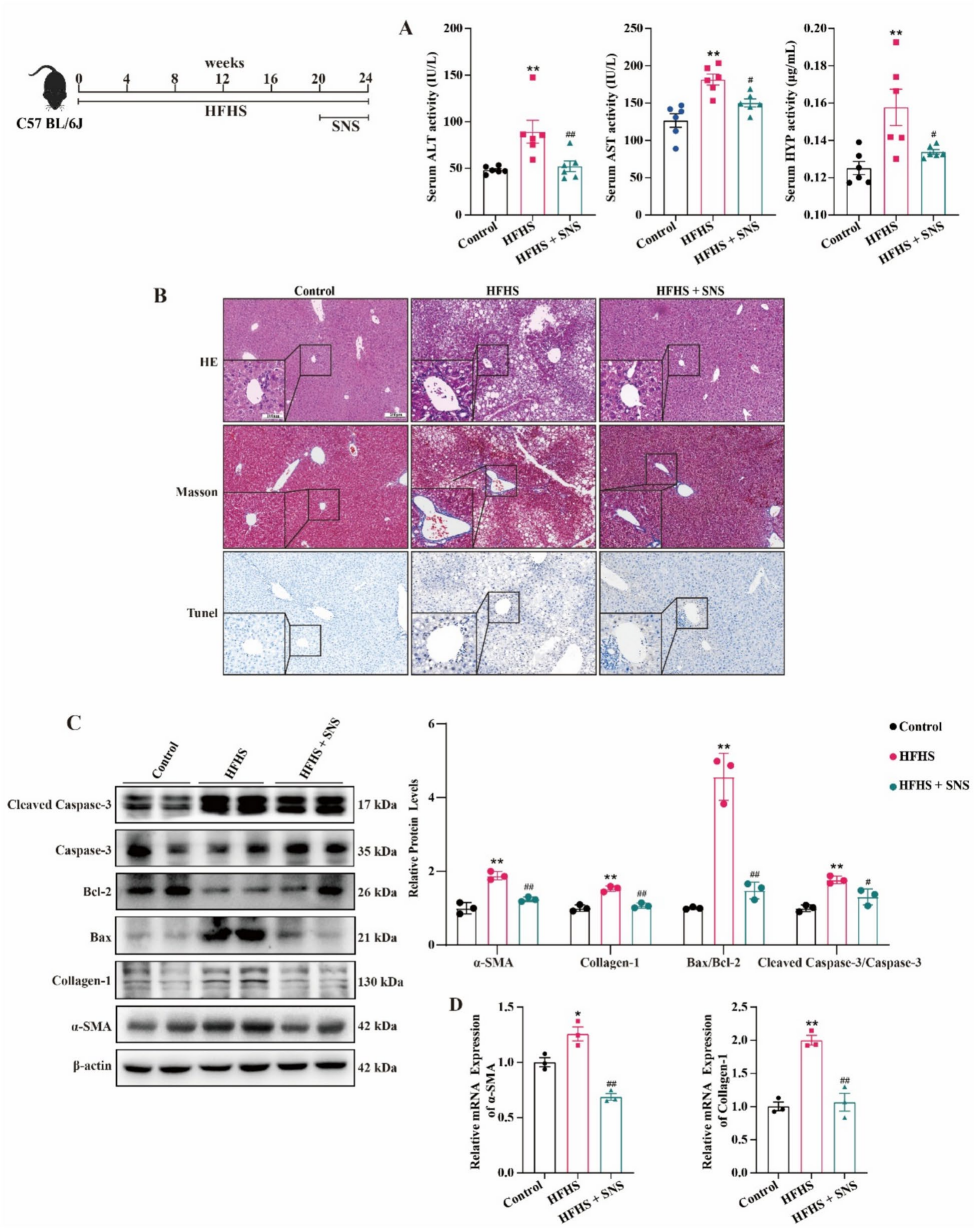
Effects of SNS on liver function, liver fibrosis and apoptosis in HFHS-induced liver fibrosis mice. **A** Serum levels of ALT, AST and HYP (n = 6). **B** HE, Masson, and TUNEL staining of liver tissue. **C** Western blotting analysis and relative quantification of α-SMA, Collagen-1, Bax, Bcl-2, cleaved Caspase-3 and Caspase-3 in liver tissue (n = 3). D The mRNA expression of *α-SMA* and *Collagen-1* was detected by RT-qPCR. (n = 3). ^*^p < 0.05, ^**^p < *0.01* VS Control group; ^*#*^*p* < *0.05, *^*##*^*p* < *0.01* VS HFHS group

In both CCl_4_- and HFHS-induced LF models, SNS treatment significantly mitigated ALT, AST and HYP levels (Fig. 1A and S2A). Meanwhile, HE staining results showed that SNS effectively reduced inflammatory cell infiltration in the liver and attenuated lipid accumulation in the HFHS model. Masson staining further confirmed SNS improved collagen deposition in liver tissue (Fig. 1B and S2B). The mRNA and protein expression levels of α-SMA and Collagen-1 similarly confirmed the protective effects of SNS against LF in different mouse models. Notably, we observed that SNS treatment reduced the protein expression ratios of cleaved Caspase-3/Caspase-3 and Bax/Bcl-2 in the liver of fibrotic mice (Fig. 1C and S2C). This result suggests an ameliorative effect of SNS on hepatocyte apoptosis, another hallmark pathological change of LF.

### SNS restores CCl_4_ or HFHS-induced BAs imbalance

**Fig. 2:**
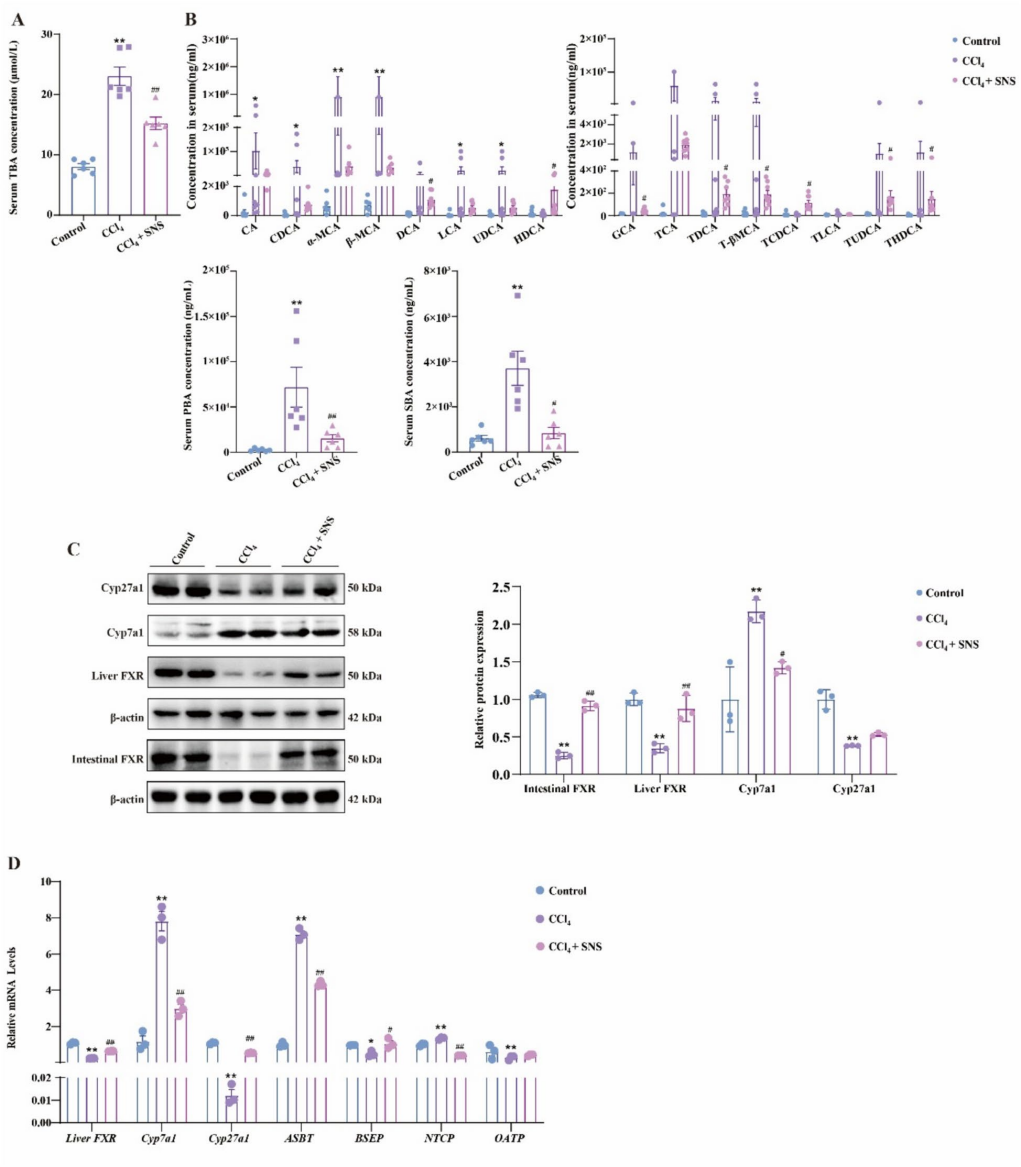
Effects of SNS on BAs metabolism in CCl_4_-induced liver fibrosis mice. **A** Serum TBA concentration (n = 6). **B** Serum BAs profile, PBAs and SBAs concentration (n = 6). **C** Western blotting analysis and relative quantification of intestinal FXR, liver FXR, Cyp7a1, Cyp27a1 (n = 3). **D** The mRNA expression of *Fxr, Cyp7a1, Cyp27a1, Asbt, Bsep, Ntcp* and *Oatp* was detected by RT-qPCR (n = 3). *p < 0.05, **p < *0.01* VS Control group; ^*#*^*p* < *0.05, *^*##*^*p* < *0.01* VS CCl_4_ group

CCl_4_ treatment increased serum total BA (TBA) levels, with elevated concentrations of primary bile acids (PBAs), including cholic acid (CA), chenodeoxycholic acid (CDCA), α-muricholic acid (α-MCA) and β-muricholic acid (β-MCA), as well as secondary bile acids (SBAs), such as lithocholic acid (LCA) and ursodeoxycholic acid (UDCA). Overall analysis revealed that both PBAs and SBAs were elevated in mice with CCl_4_-induced liver fibrosis. SNS treatment significantly reduced these aberrant BAs levels (Fig. 2A, B). Furthermore, CCl_4_ exposure led to the downregulation of Cyp7a1, a key enzyme in BAs biosynthesis, while upregulating Cyp27a1, and decreased FXR protein expression in both the liver and intestine. Additionally, CCl_4_ disrupted the mRNA expression of BAs transporters, including *Asbt, Bsep, Ntcp* and* Oatp*. SNS treatment showed a trend toward improving Cyp27a1 expression, though the change was not statistically significant. Notably, SNS significantly restored the expression of CYP7a1, FXR, ASBT, and other BAs transporters. (Fig. 2C, D).

**Fig. 3:**
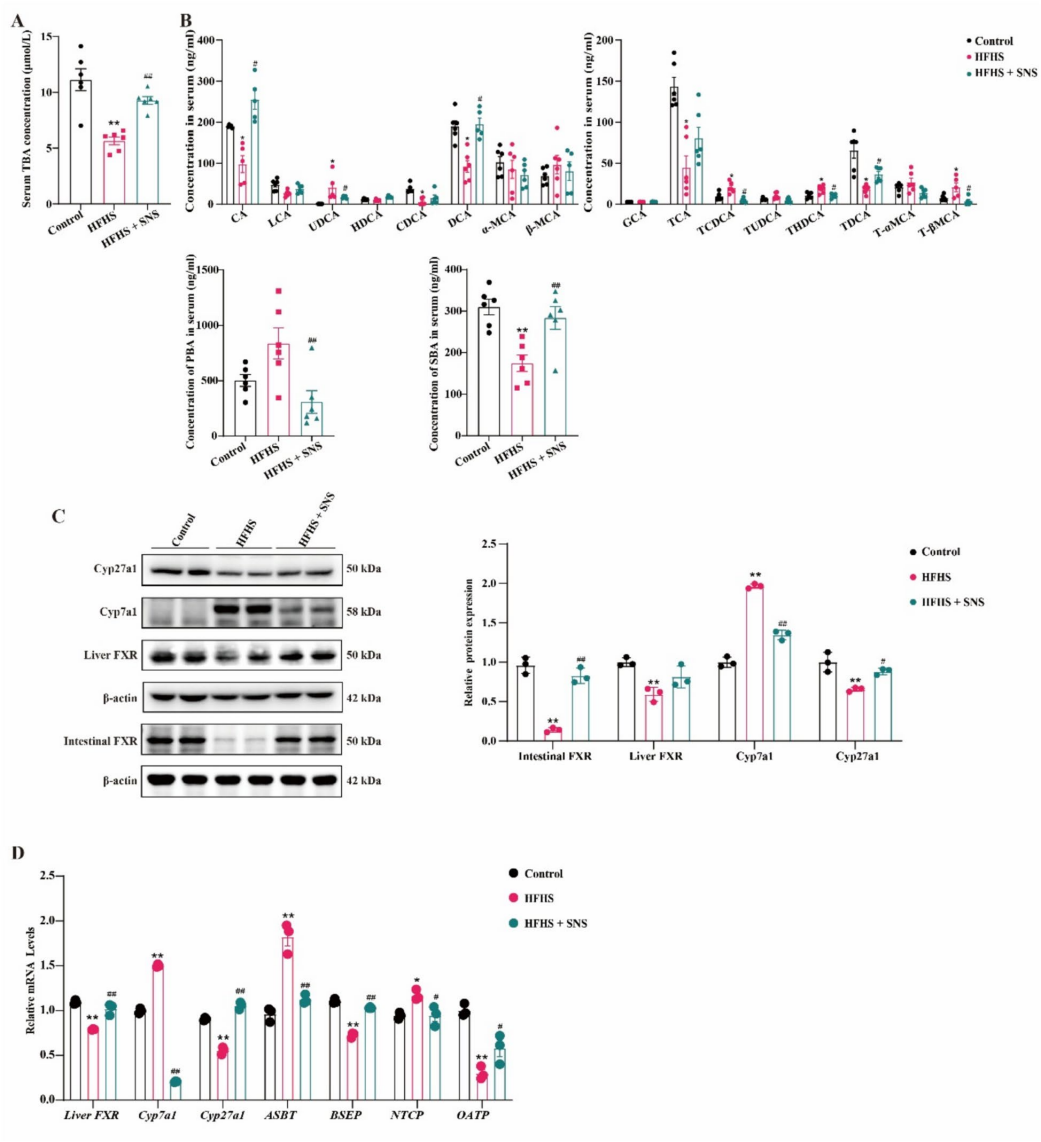
Effects of SNS on BAs metabolism in HFHS-induced liver fibrosis mice. **A** Serum TBA concentration (n = 6). **B** Serum BAs profile, PBAs and SBAs concentration (n = 6). **C** Western blotting analysis and relative quantification of intestinal FXR, liver FXR, Cyp7a1, Cyp27a1 (n = 3). **D** The mRNA expression of *Fxr, Cyp7a1, Cyp27a1, Asbt, Bsep, Ntcp* and *Oatp* was detected by RT-qPCR (n = 3). ^*^p < 0.05, ^**^p < *0.01* VS Control group; ^*#*^*p* < *0.05, *^*##*^*p* < *0.01* VS HFHS group

In contrast, HFHS reduced TBA levels, with PBAs showing decreased CA, CDCA, and taurocholic acid (TCA) but increased taurochenodeoxycholic acid (TCDCA) and tauro-β-muricholic acid (T-βMCA). SBAs exhibited decreased deoxycholic acid (DCA) and taurochenodeoxycholic acid (TDCA), while UDCA and taurohyodeoxycholic acid (THDCA) levels increased. Overall, in mice with HFHS-induced liver fibrosis, PBAs were elevated, whereas SBAs were decreased. SNS treatment significantly alleviated these alterations (Fig. 3A, B). Similar to the CCl_4_-induced LF model, SNS modulated BAs synthesis, transport, and metabolism in HFHS-induced LF mice. Although SNS restored hepatic FXR expression, no statistically significant difference was observed; however, it significantly enhanced FXR expression in the intestine. (Fig. 3C, D). These findings suggest that SNS effectively restores BAs homeostasis in both CCl_4_ and HFHS-induced LF models.

### SNS restores CCl_4_ and HFHS-induced intestinal flora disorders

16S rRNA sequencing was performed on mouse fecal samples. α-diversity and β-diversity analyses revealed distinct clustering patterns among the three groups. The LF group displayed shifts in the abundance and diversity of the intestinal microbiota, which SNS treatment partially restored (Fig. S3a, b).

**Fig. 4:**
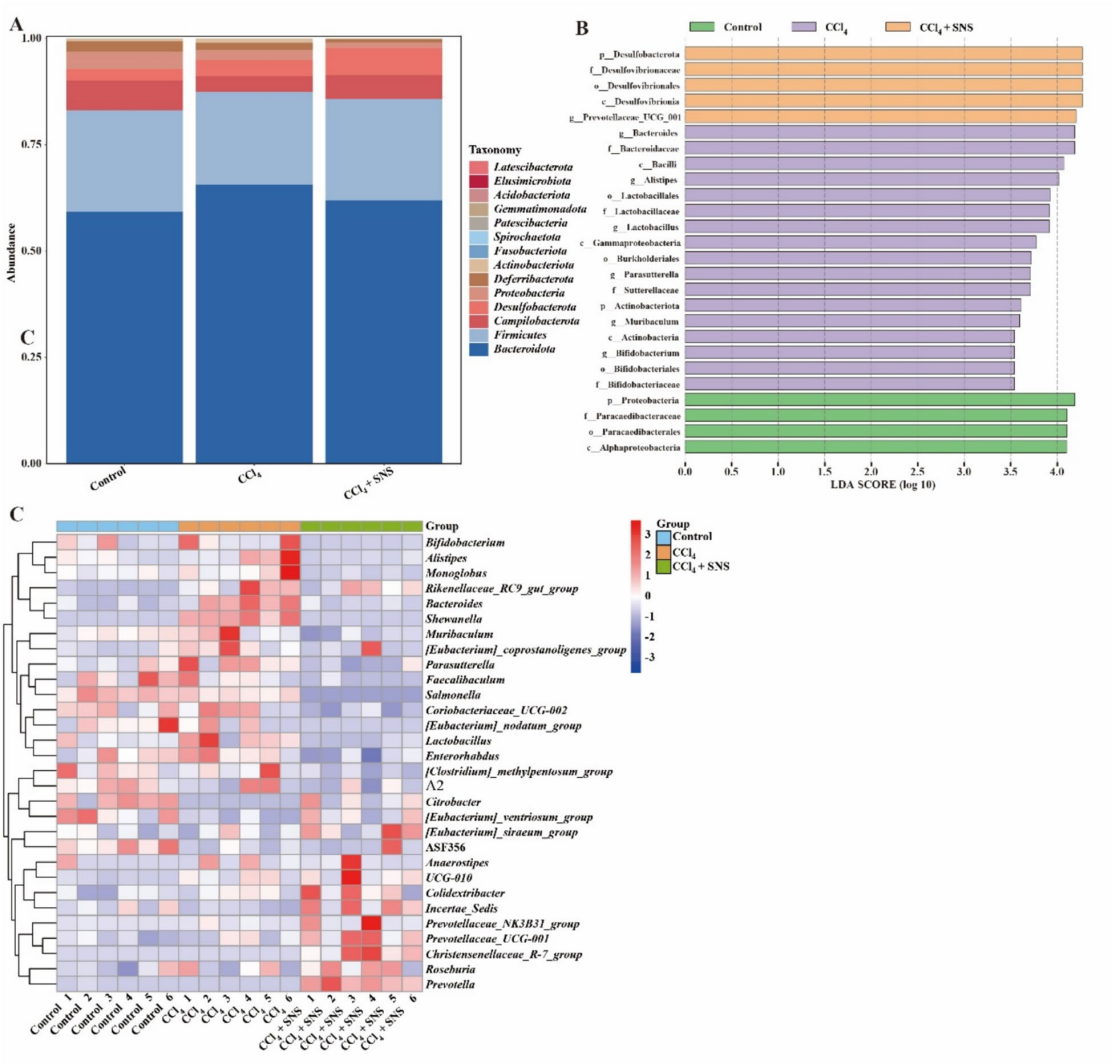
Effects of SNS on the relative abundance of intestinal microbiota and BA metabolism in CCl_4_-induced liver fibrosis mice (n = 6). **A** Taxonomic composition at the phylum level for the 15 most abundant intestinal microbiota. **B** Linear discriminant analysis (LDA) scores derived using LEfSe to identify differentially enriched features among the three groups. Different colors denote distinct groups, with features selected based on LDA scores > 3.0. **C** Heatmap showing differential abundance of genera

**Fig. 5:**
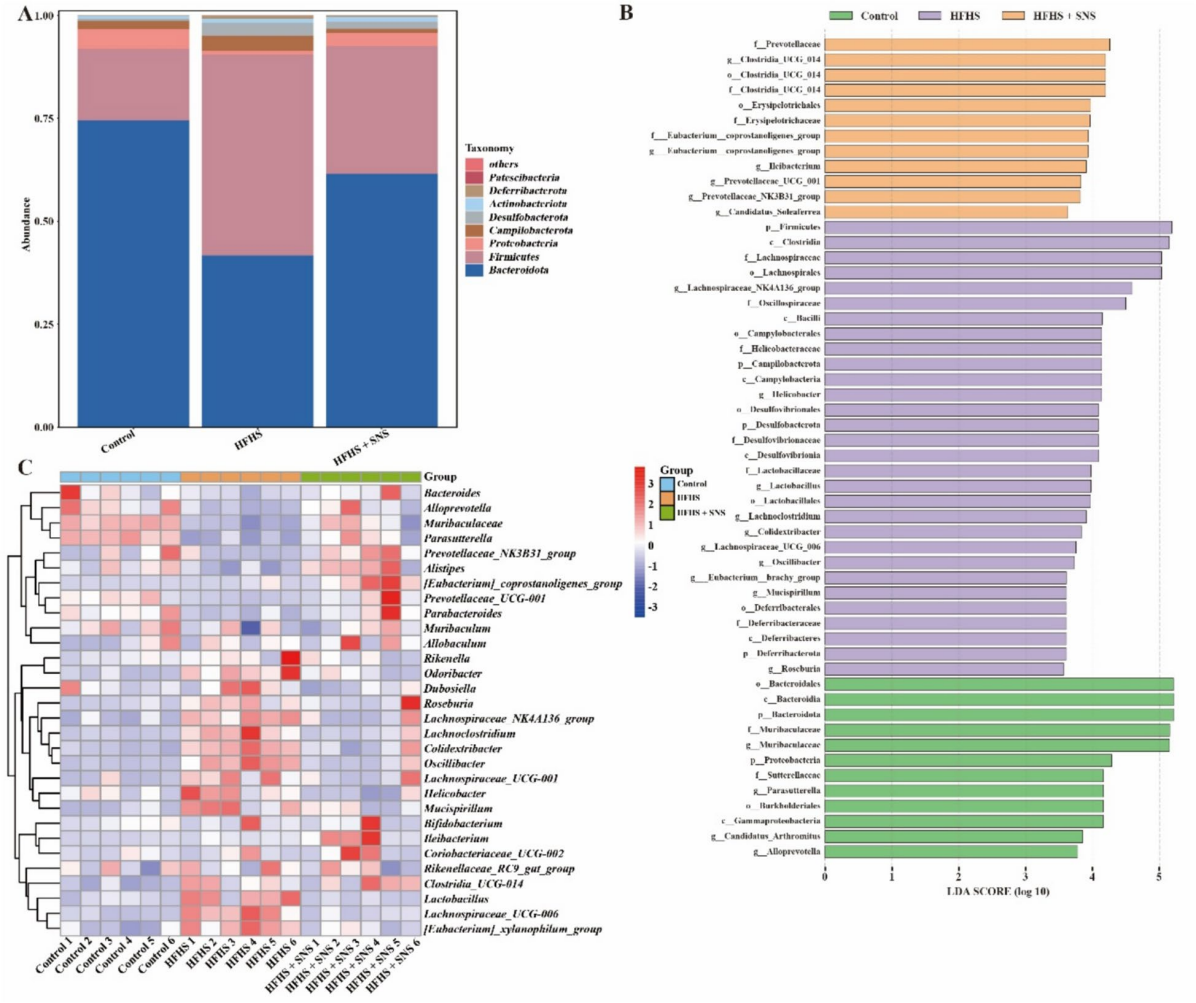
Effects of SNS on the relative abundance of intestinal microbiota and BA metabolism in HFHS-induced liver fibrosis mice (n = 6). **A** Taxonomic composition at the phylum level for the 15 most abundant intestinal microbiota. **B** Linear discriminant analysis (LDA) scores derived using LEfSe to identify differentially enriched features among the three groups. Different colors denote distinct groups, with features selected based on LDA scores > 3.0. **C** Heatmap showing differential abundance of genera

In the CCl_4_-induced LF model, SNS treatment restored microbial imbalances at the phylum level, where *Bacteroidota* was elevated, while *Firmicutes* and *Campilobacterota* were reduced (Fig. 4A). At the genus level, SNS treatment normalized the altered abundances of *Bacteroides*, *Lactobacillus*, *Parasutterella*, and *Muribaculum* (Fig. 4B, C). In the HFHS-induced LF model, SNS treatment corrected microbial dysbiosis, reversing the decreased relative abundance of *Bacteroidota* and *Proteobacteria* and the increased abundance of *Firmicutes*, *Campilobacterota* and *Desulfobacterota* at the phylum level (Fig. 5A). At the genus level, SNS modulated the abundances of *Lachnospiraceae_NK4A136_group*, *Helicobacter*, *Lactobacillus*, *Lachnoclostridium*, *Colidextribacter*, *Muribaculaceae*, *Parasutterella*, *Bacteroides*, and *Prevotellaceae_UCG-001* (Fig. 5B, C). Overall, SNS effectively restored gut microbial homeostasis by improving microbial diversity and composition in both LF models.

**Fig. 6:**
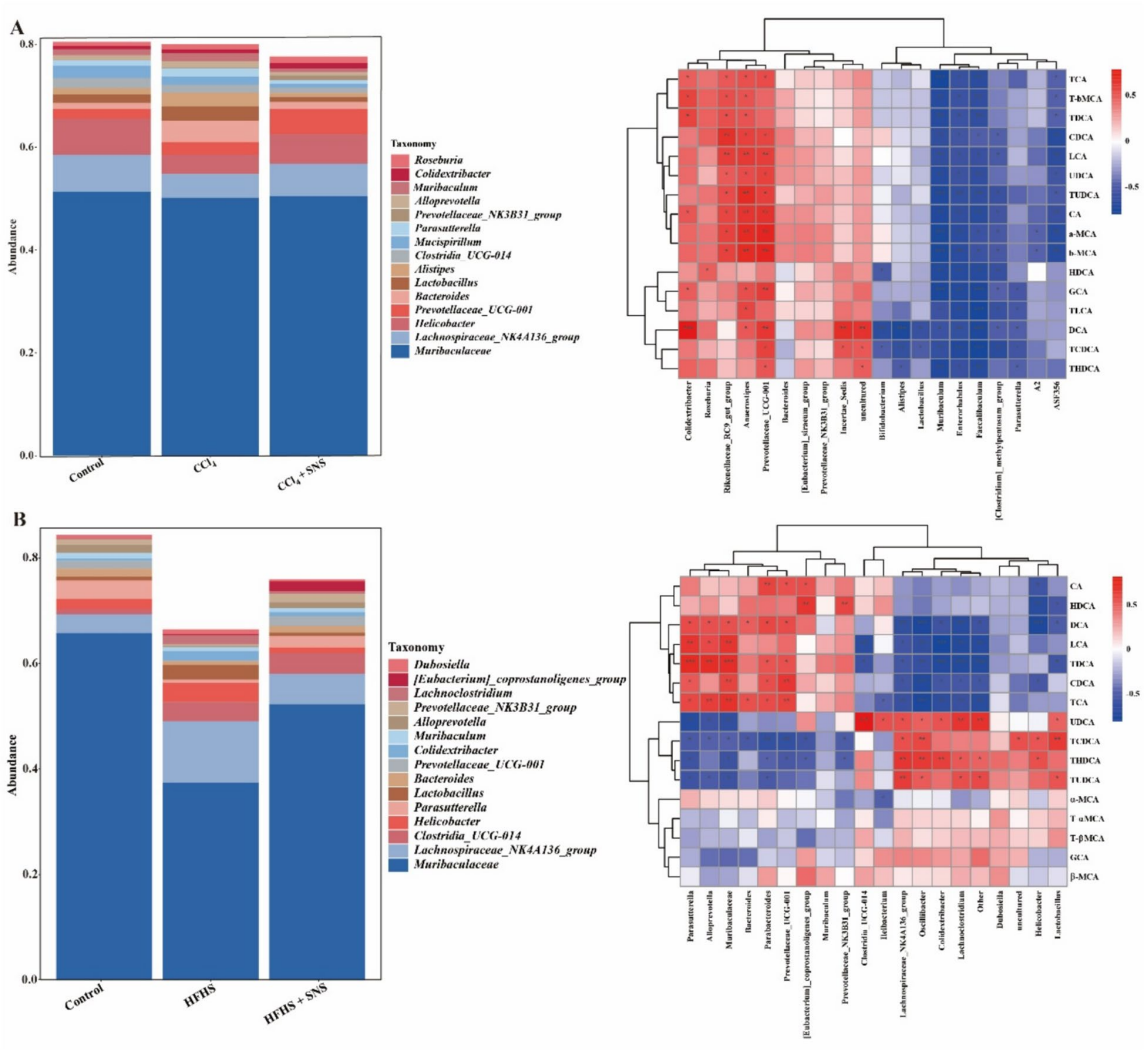
Correlation analysis of serum BA levels and intestinal microbiota in CCl_4_ and HFHS-induced liver fibrosis mice. **A** Genus-level taxonomic composition of the top 15 dominant microbiota in CCl_4_-induced liver fibrosis mice, along with correlation analysis results. **B** Genus-level taxonomic composition of the top 15 dominant microbiota in HFHS-induced liver fibrosis mice, along with correlation analysis results. Data were obtained from an independent experiment (n = 6). ^*^p < *0.05*

We then analyzed the correlation between these flora and BAs metabolism. In the CCl_4_-induced LF model, *Lactobacillus* was negatively correlated with DCA and TCDCA, *Parasutterella* was negatively correlated with glycocholic acid (GCA), taurolithocholic acid (TLCA), DCA, TCDCA, and THDCA, and *Muribaculum* was inversely correlated with all detected BAs (Fig. 6A). In the HFHS-induced LF model, *Lachnospiraceae_NK4A136_group* was positively correlated with THDCA and TCDCA, *Helicobacter* with TCDCA and tauroursodeoxycholic acid (TUDCA), and *Lachnoclostridium* with UDCA. *Parasutterella* was positively correlated with DCA, hyodeoxycholic acid (HDCA), CDCA, CA, GCA, and TDCA, while *Muribaculaceae* had a negative correlation with UDCA, TCDCA, THDCA, and TUDCA, and was positively correlated with DCA, LCA, TDCA, CDCA, and TCA (Fig. 6B). We analyzed the effects of SNS on gut microbiota associated with BAs levels and found that SNS treatment modulated their dysregulated relative abundances. This highlights its role in shaping gut microbiota composition, regulating BAs metabolism, and mitigating LF progression (Figure S4).

### Cholestyramine antagonizes the antifibrotic effects of SNS in LF mice

**Fig. 7:**
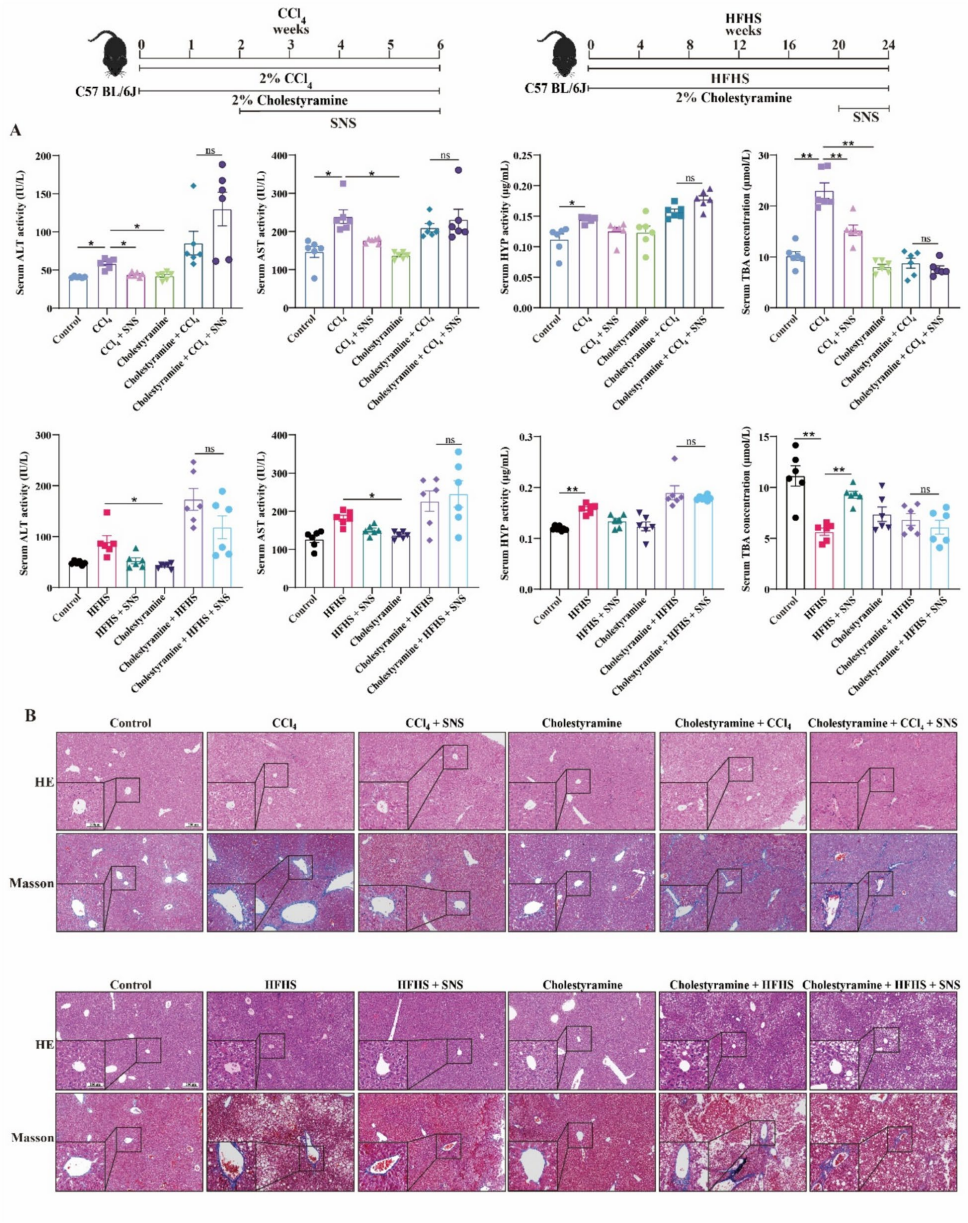
Effects of SNS on liver function and liver fibrosis in CCl_4_ and HFHS-induced liver fibrosis mice following cholestyramine-mediated chelation of BAs. **A** Serum levels of ALT, AST, HYP and TBA (n = 6). **B** HE and Masson staining of liver tissue (n = 3). ^*^p < *0.05*

**Fig. 8:**
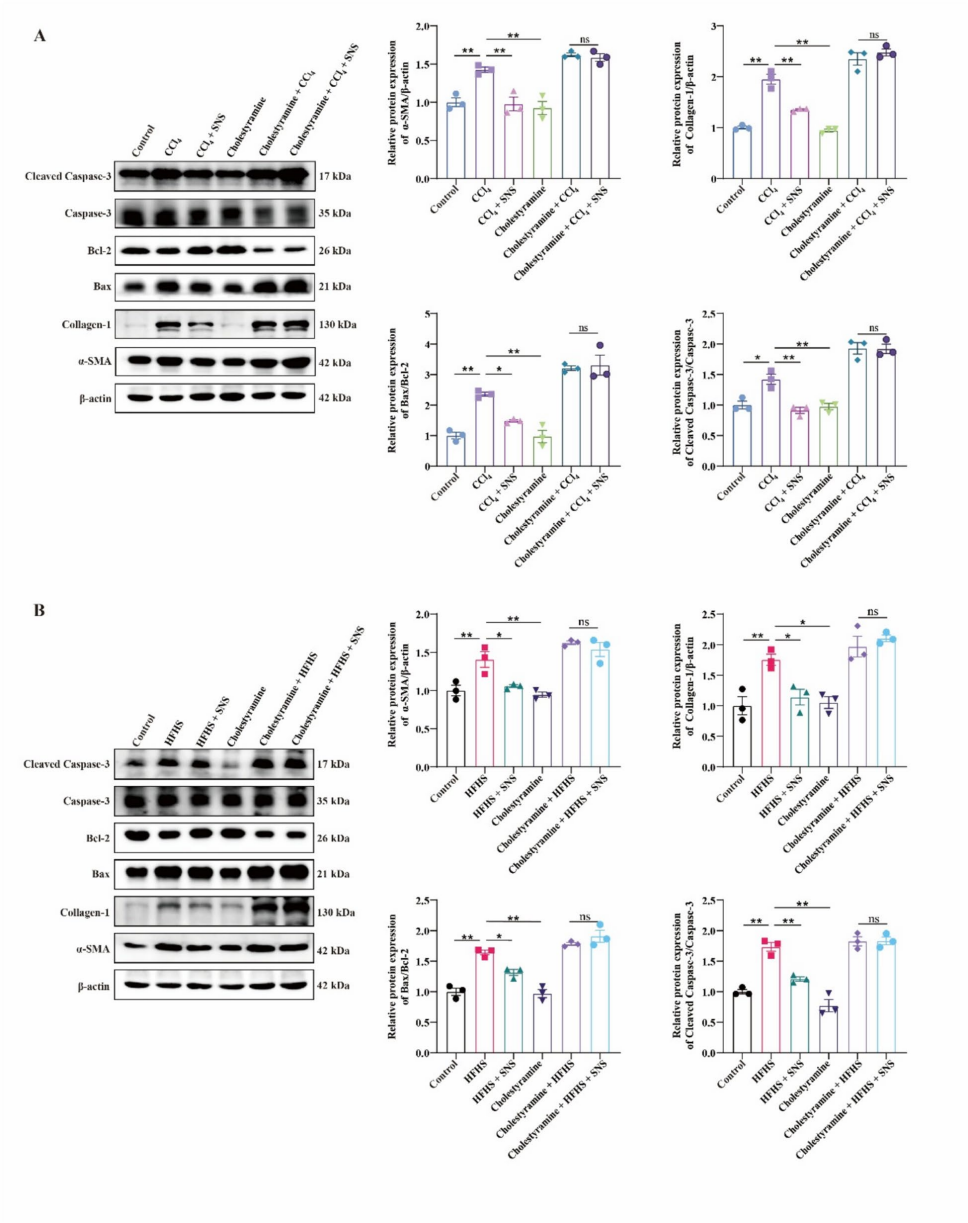
Effects of SNS on liver fibrosis and apoptotic protein expression in CCl_4_ and HFHS-induced liver fibrosis mice following cholestyramine-mediated chelation of BAs. **A** Western blotting analysis and relative quantification of α-SMA, Collagen-1, Bax, Bcl-2, cleaved Caspase-3 and Caspase-3 in CCl_4_-induced liver fibrosis model (n = 3). **B** Western blotting analysis and relative quantification of α-SMA, Collagen-1, Bax, Bcl-2, cleaved Caspase-3 and Caspase-3 in HFHS-induced liver fibrosis model (n = 3). ^*^p < *0.05*

To assess the impact of BAs homeostasis on LF progression, Cholestyramine was used to chelate BAs. While Cholestyramine had no effect on ALT, AST and HYP levels in Control group, it markedly elevated ALT, AST and HYP levels in CCl_4_- and HFHS-induced LF models (Fig. 7A). HE and Masson staining revealed that pathological damage, including liver inflammation, fibrosis, and lipid deposition, was exacerbated following Cholestyramine-induced BAs chelation in LF mice (Fig. 7B). The protein expression of α-SMA, Collagen-1, Bax/Bcl-2, and Cleaved Caspase-3/Caspase-3 in liver tissues further supported these observations (Fig. 8A, B), indicating that disruption of BAs homeostasis exacerbated fibrosis in LF model mice. Notably, Cholestyramine abolished the protective effects of SNS on liver injury, fibrosis, and hepatocyte apoptosis. These results suggest that BAs homeostasis may be a critical target of SNS in preventing LF.

### Antibiotic cocktail inhibits the antifibrotic efficacy of SNS in LF mice

**Fig. 9:**
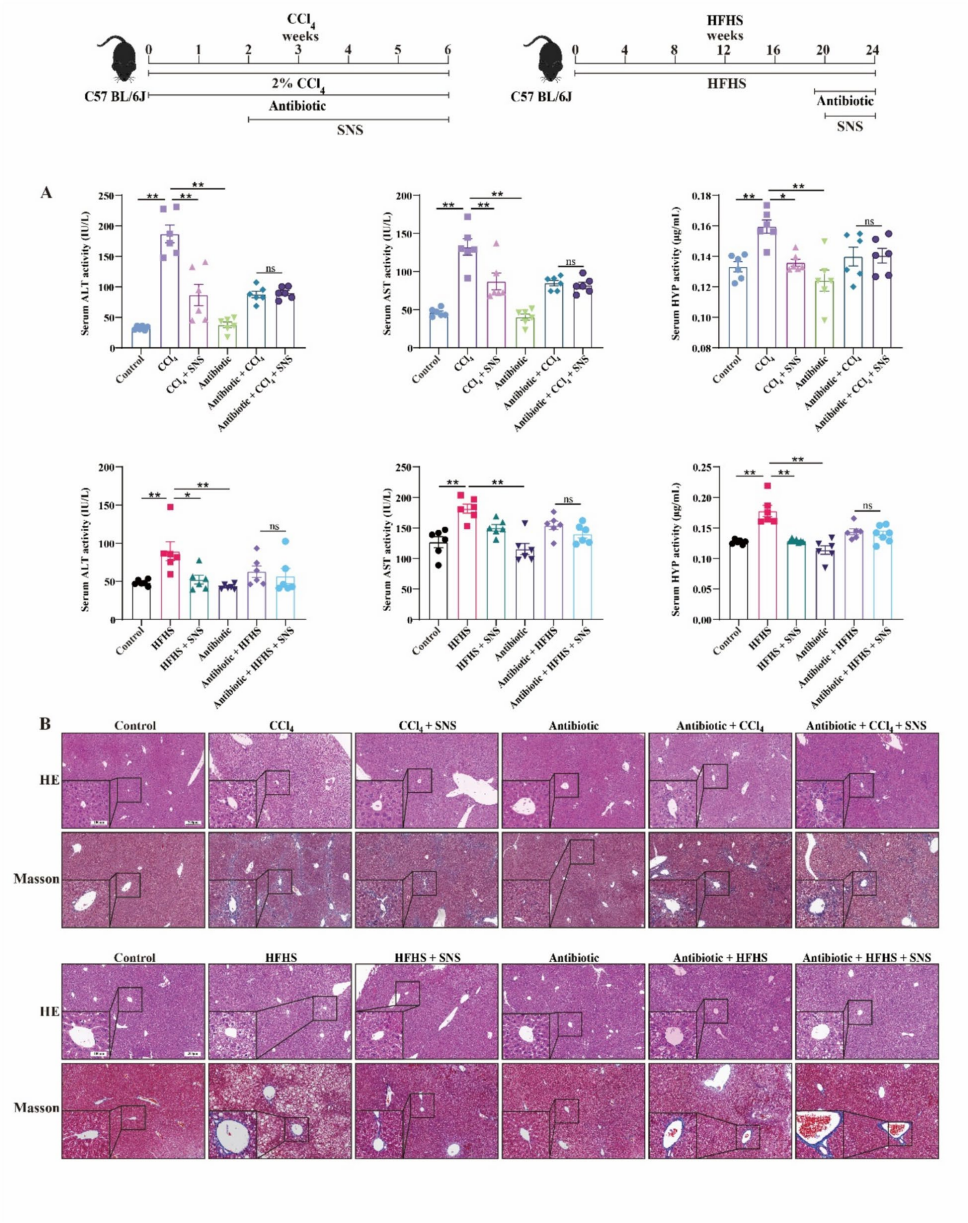
Effects of SNS on liver function and fibrosis in CCl_4_ and HFHS-induced liver fibrosis model in pseudo-sterile mice. **A** Serum levels of ALT, AST and HYP (n = 6). **B** HE and Masson staining of liver tissue (n = 3). ^*^p < *0.05*

**Fig. 10:**
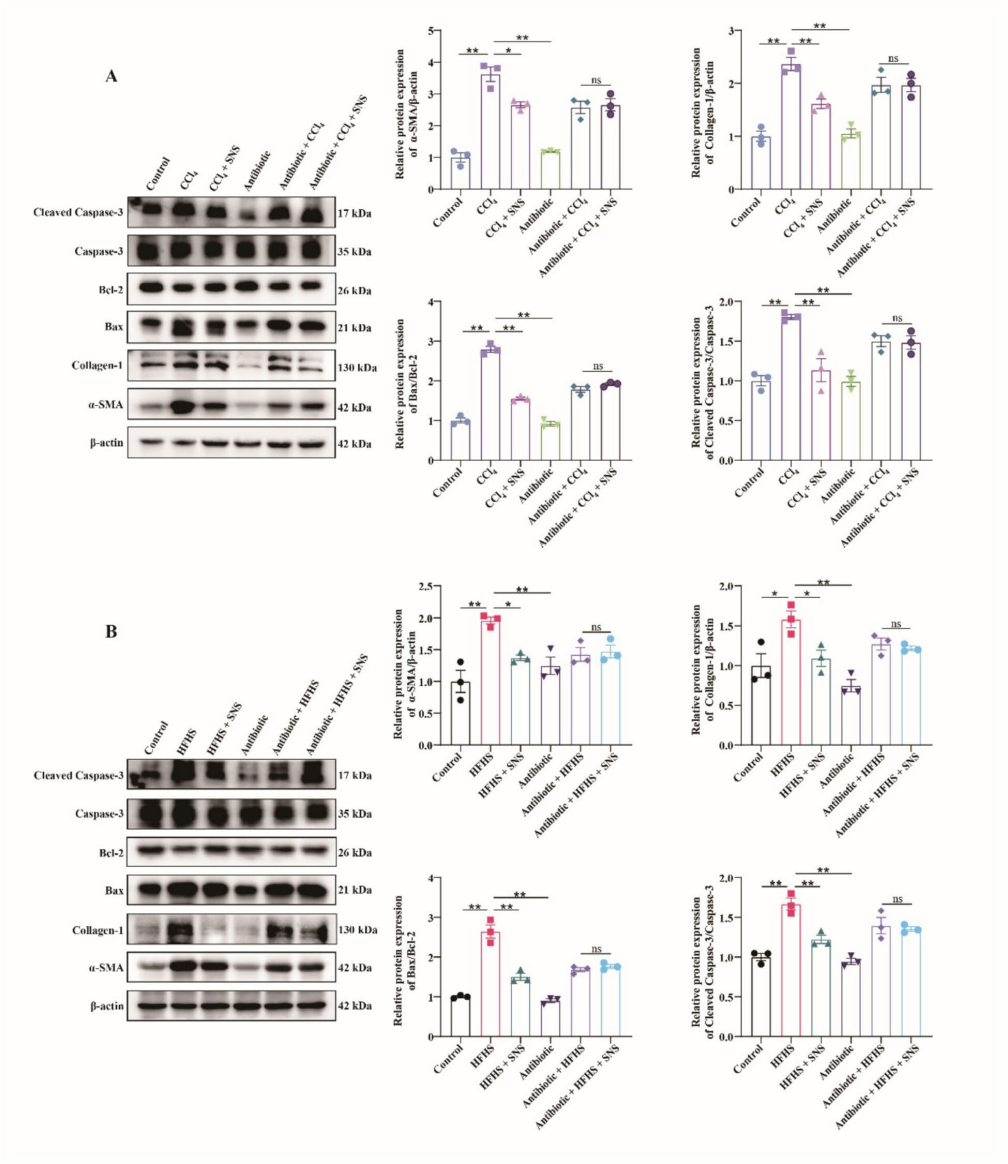
Effects of SNS on liver fibrosis and apoptotic protein expression in CCl_4_ and HFHS-induced liver fibrosis model in pseudo-sterile mice. **A** Western blotting analysis and relative quantification of α-SMA, Collagen-1, Bax, Bcl-2, cleaved Caspase-3 and Caspase-3 in CCl_4_-induced liver fibrosis model (n = 3). **B** Western blotting analysis and relative quantification of α-SMA, Collagen-1, Bax, Bcl-2, cleaved Caspase-3 and Caspase-3 in HFHS-induced liver fibrosis model (n = 3). ^*^p < *0.05*

To investigate whether SNS regulates BAs homeostasis through gut microbiota, we established a pseudo-sterile mouse model via broad-spectrum antibiotic treatment. Serum ALT, AST and HYP levels, along with HE and Masson staining, demonstrated that the ameliorative effect of SNS on CCl_4_ and HFHS-induced LF models was abolished in pseudo-sterile mice (Fig. 9A, B). Protein expression of α-SMA, Collagen-1, Bax/Bcl-2, and Cleaved Caspase-3/Caspase-3 further confirmed these observations (Fig. 10A, B). Analysis of TBA and BAs in serum from pseudo-sterile mice revealed that SNS failed to correct BAs imbalance after antibiotic treatment (Figs. S5 and S6). These results suggest that SNS may regulate BAs homeostasis by restoring gut microbiota balance, underscoring the role of gut flora in ameliorating LF.

### FXR knockout attenuates the antifibrotic efficacy of SNS in LF mice

**Fig. 11:**
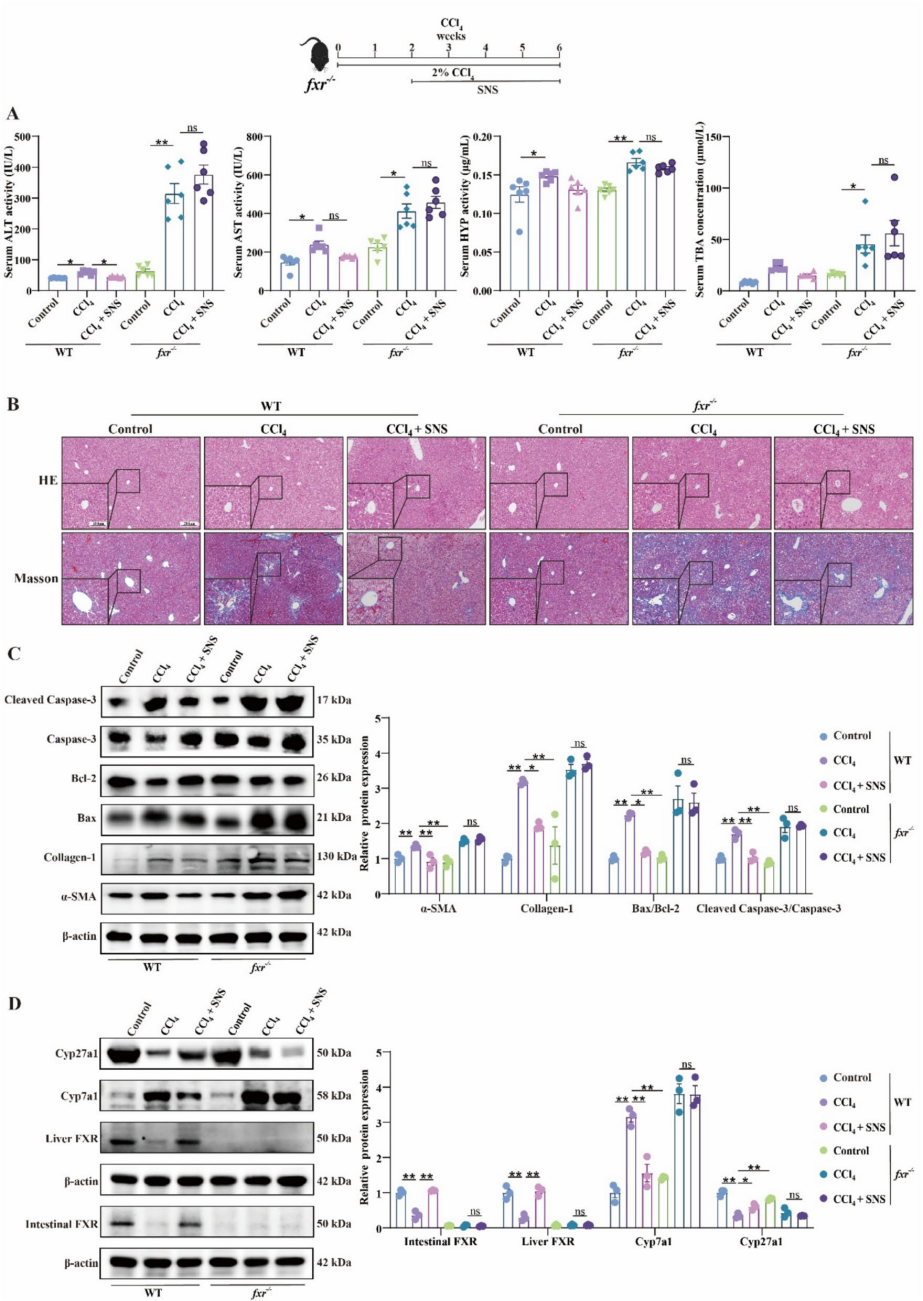
Effects of SNS on liver function, liver fibrosis and apoptosis in *fxr*^*−/−*^ mice. **A** Serum levels of ALT, AST, HYP and TBA. (n = 6). **B** HE and Masson staining of liver tissue (n = 3). **C** Western blotting analysis and relative quantification of α-SMA, Collagen-1, Bax, Bcl-2, cleaved Caspase-3 and Caspase-3 (n = 3). **D** Western blotting analysis and relative quantification of intestinal FXR, liver FXR, Cyp7a1, Cyp27a1 (n = 3). ^*^p < *0.05*

FXR-mediated negative feedback regulation of BAs is essential for maintaining BAs homeostasis. We further explored whether SNS modulates BAs metabolism through FXR. FXR knockdown exacerbated CCl_4_-induced liver injury, as evidenced by elevated AST ALT and HYP levels, increased inflammatory infiltration, and collagen deposition in the liver (Fig. 11A, B). The ameliorative effect of SNS on liver injury and fibrosis observed in wild-type mice was abolished in *fxr*^*−/−*^ mice, as demonstrated by western blot analysis (Fig. 11C). Western blot results also confirmed the deletion of FXR in both the liver and intestine. Following FXR knockout, hepatic Cyp7a1 expression was upregulated compared with wild-type mice, while Cyp27a1 expression showed no significant change (Fig. 11D). These alterations corresponded with the marked increase in TBA levels in CCl_4_-induced LF in *fxr*^*−/−*^ mice, further confirming the pivotal role of FXR in BAs homeostasis. Notably, SNS treatment failed to normalize the elevated TBA levels or the aberrant expression of synthetic enzymes in *fxr*^*−/−*^ mice. These results suggest a critical role for FXR in SNS regulation of BAs homeostasis.

## Discussion

SNS is a traditional Chinese medicine formula extensively applied in the treatment of various liver diseases, including LF. Our previous studies have confirmed its efficacy in mice with CCl_4_-induced LF. However, its pharmacological mechanism remains unclear, and its therapeutic effects under different etiological conditions have not been systematically evaluated. To broaden the scope of investigation, we introduced a HFHS diet-induced fibrosis model in addition to the classical CCl_4_ model. The HFHS model closely simulates the metabolic disturbances, chronic inflammation, and progressive fibrogenesis observed in patients with NAFLD, a major and increasingly prevalent cause of liver fibrosis worldwide [22]. The combination of these two models enabled us to assess the effects of SNS across distinct pathological contexts and to provide a more comprehensive basis for elucidating its potential mechanisms. The results showed that SNS alleviated hepatic fibrosis and improved BAs homeostasis dysregulation in both models, suggesting that its therapeutic effects may be associated with the modulation of BAs homeostasis.

The homeostasis of BAs relies on the dynamic stability of the BAs pool, as even minor alterations in BAs levels or composition can significantly affect liver physiological processes. CA, a primary bile acid, has been reported to exacerbate liver fibrosis in rats fed a high-fat, high-cholesterol (HFC) diet [23]. In addition, while secondary BAs exhibit anti-inflammatory properties, their excessive accumulation can induce hepatic inflammation [24, 25]. Additionally, TCA promotes hepatic stellate cell activation and LF through the S1PR2/p38 MAPK/YAP signaling pathway [26]. Similarly, GCDCA activates the epidermal growth factor receptor/MEK/ERK and p38/JNK pathways, leading to hepatic stellate cell proliferation and exacerbation of LF [27]. Activation of the endogenous BAs receptor FXR mitigates LF by reducing BAs synthesis via a negative feedback mechanism and enhancing their excretion. Conversely, secondary BAs, such as DCA and LCA, activate TGR5, which triggers ERK1/2 and JNK signaling pathways, exerting anti-inflammatory and anti-fibrotic effects [4]. These findings underscore the intricate roles of various BAs types and receptors in LF progression. Thus, maintaining BAs levels and composition within physiological ranges is crucial for developing effective therapeutic strategies for LF.

BAs analogs and receptor-targeting therapies have demonstrated considerable potential in the treatment of liver diseases. UDCA, one of the earliest BA-derived drugs, is widely employed for managing primary biliary cholangitis (PBC) and cholestatic liver diseases. The gut-specific FXR antagonist GβMCA reduces the BA pool size and hydrophobicity, thereby ameliorating LF and improving intestinal barrier function in Cyp2c70 KO mice [28]. In 2016, the FXR agonist OCA was approved by the US Food and Drug Administration for the treatment of PBC. However, its application is associated with a high incidence of adverse effects [29]. Interestingly, combining OCA with the apoptosis inhibitor IDN-6556 alleviated LF by modulating BA homeostasis and enhancing antifibrotic effects [18]. These findings suggest that multi-target approaches regulating BAs homeostasis may be essential for achieving optimal therapeutic outcomes in LF management.

In this study, serum TBA levels were elevated in the CCl_4_-induced LF model group but decreased in the HFHS model group. Classification analysis of the BAs pool composition revealed increased levels of PBAs in both models, accompanied by a decrease in SBAs in the HFHS group. In addition, activation of the classical BA synthesis pathway, with Cyp7a1 as the rate-limiting enzyme, was observed. This pathway converts cholesterol into PBAs, which is consistent with the elevated PBAs levels detected in both fibrosis models. Moreover, FXR expression in both the liver and intestine was downregulated, a change that typically indicates reduced BA secretion and enhanced reabsorption [7]. Although the expression patterns of BAs metabolism-related molecules were comparable in both models, the differences in serum BAs levels and composition might be attributed to increased BAs excretion in high-fat diet-fed mice [30]. In addition, given that the generation of SBAs is closely associated with gut microbiota activity, the decline in BAs content observed in the HFHS group may also result from microbiota alterations induced by the specific dietary pattern. Notably, the two LF models displayed distinct intestinal flora profiles. These findings highlight the complexity of BAs dysregulation and suggest that BAs homeostasis is influenced by multiple factors. In mice with CCl_4_ and HFHS-induced fibrosis, SNS treatment effectively restored BAs homeostasis.

Subsequently, intestinal BAs were chelated using cholestyramine, which facilitates BAs excretion through feces. Cholestyramine treatment further aggravated liver injury in both LF models, and the therapeutic effects of SNS on LF were completely abrogated following BAs chelation. These findings highlight the significance of BAs homeostasis in LF progression and its contribution to the pharmacological mechanisms of SNS.

The gut microbiota plays a fundamental role in BAs metabolism and regulates BAs-mediated signal transduction processes. In the intestine, PBAs are biotransformed into SBAs by microbial enzymes through reactions such as deconjugation, dehydroxylation, oxidation and epimerization, esterification, and de-esterification [31]. Numerous studies have documented alterations in intestinal flora during LF, which are closely linked to disruptions in BAs homeostasis [32]. For instance, in patients with NAFLD, LF is characterized by elevated serum GCA and fecal DCA levels, which show positive correlations with *Lachnospiraceae* and negative correlations with *Bacteroidaceae* [33]. Similarly, in HBV-induced LF, fecal primary and secondary BAs exhibit negative correlations with *Ruminococcus* and *Escherichia*, respectively [34]. Previous studies have also demonstrated that in mice with liver fibrosis, SBAs biosynthesis and bile salt hydrolase (BSH) activity are markedly elevated. Moreover, the relative abundance of several bacterial genera and species—including *Clostridium, Bacteroides, Lactococcus, Streptococcus, Escherichia, Pseudomonas* and *Fusobacterium*—was significantly increased. Notably, these microbial taxa showed strong positive correlations with the fecal concentrations of specific BAs, such as DCA, TDCA, GDCA, and TCA, as well as CDCA, UDCA, LCA, TCDCA, TUDCA, GCDCA, and GLCA [35]. In this study, we identified significant alterations in the composition of intestinal flora in CCl_4_ and HFHS-induced LF models, with several of these changes closely linked to BAs metabolism. For instance, *Lactobacillus* is involved in BAs dissociation, while *Lachnospiraceae* contributes to the conversion of secondary BAs [36]. We noticed that the gut microbiota composition differed between the CCl_4_ and HFHS induced liver fibrosis models, which might partly explain the potential differences in the mechanisms by which SNS exerts its effects in these two models. Previous studies have also confirmed this distinction. In the CCl_4_ model, microbial alterations are more closely associated with the inflammation/oxidative stress axis, and inflammation-related taxa such as *Proteobacteria* have been reported to increase in several studies [37]. In contrast, gut microbiota changes in the HFHS model are mainly linked to disturbances in lipid and bile acid metabolism, typically characterized by an increased ratio of *Firmicutes* to *Bacteroidetes*, along with local changes in *Actinobacteria* or *Proteobacteria* [38]. In addition, we specifically noted that the genus *Parasutterella* exhibited a similar trend of change in both fibrosis models, which might suggest a shared microbial signature associated with liver fibrosis progression and SNS intervention. *Parasutterella* plays a role in BAs metabolism and homeostasis by regulating the expression of genes related to ileal BAs transporters and the FXR signaling pathway [39]. Notably, the relative abundance of *Parasutterella* exhibited opposite trends in CCl_4_- and HFHS-induced LF mice, suggesting an environment-dependent function under different disease states. For instance, *Parasutterella* abundance increases in chronic colitis [40] but declines in NAFLD-induced LF models, with a further decrease as fibrosis progresses [41]. This pattern aligns with our observation of reduced *Parasutterella* abundance in HFHS-induced LF mice. SNS treatment effectively reversed gut microbiota dysbiosis associated with BA metabolism, restoring microbial composition. These findings suggest that SNS exerts its therapeutic effects on LF by modulating gut microbiota to maintain BAs homeostasis. Furthermore, the loss of SNS efficacy in pseudo-sterile mice underscores the essential role of gut microbiota in mediating this process.

In addition to the gut microbiota, BAs synthesis and metabolic homeostasis are regulated through negative feedback by FXR, a BA-activated nuclear receptor. FXR activation suppresses the transcription of BA-metabolizing enzymes, such as CYP7A1 and CYP8B1, in the liver and intestine [42]. Consistently, our results showed that CYP7A1 levels were elevated in FXR knockout mice, confirming the critical regulatory role of FXR in controlling CYP7A1 expression [7]. Both hepatic and intestinal FXR contribute to the maintenance of BA homeostasis [43, 44]. In fibrotic mice, expression of FXR in both tissues was decreased, accompanied by dysregulation of downstream targets under FXR-mediated negative feedback control, including Cyp7a1, ASBT, NTCP, BSEP and OATP. Furthermore, liver fibrosis induction in FXR-deficient mice revealed that FXR ablation aggravated BAs dysregulation and accelerated LF progression [45], consistent with our research. Moreover, the therapeutic effects of SNS on LF were completely absent in *fxr*^*−/−*^ mice, underscoring the critical role of FXR in mediating the efficacy of SNS in alleviating LF.

We observed that SNS restored BAs homeostasis through the modulation of gut microbiota and FXR expression. We observed that SNS restored bile acid homeostasis by modulating gut microbiota and FXR expression; however, its underlying mechanisms may differ between the CCl_4_ and HFHS induced liver fibrosis models. These differences might, on one hand, stem from the intrinsic variations in gut microbial composition between the two models as mentioned earlier, and on the other hand, the diverse bioactive components of SNS may exert selective effects on different bacterial taxa. For instance, hesperidin has been shown to ameliorate obesity, intestinal barrier dysfunction, and inflammation in high-fat diet models by increasing the abundance of Lactobacillus and IgA-coated bacteria [46]. Similarly, paeoniflorin has been reported to improve gut microbial composition and enhance probiotic abundance in depression-related models, mitigating dysbiosis-induced host effects [47]. Moreover, paeoniflorin treatment also exerts protective effects via the microbiota–metabolite axis in colitis models [48].

We acknowledge that elucidating the precise mechanisms of these bioactive components remains one of the limitations of the present study, and the specific bacterial taxa targeted by SNS have yet to be identified. Further investigation using microbiota transplantation experiments will be necessary to clarify this issue. In addition, we were unable to establish an HFHS-induced LF model in *fxr*^−/−^ mice, as these mice spontaneously develop liver injury at approximately 6 months of age, characterized by lipid accumulation, vacuolation, and focal necrosis, progressing to hepatocellular carcinoma by 15 months [49, 50]. Given that our modeling period lasted 24 weeks, it was difficult to ensure the stability of the HFHS-induced LF model in *fxr*^*−/−*^ mice. Moreover, while our findings indicate that SNS regulates bile acid homeostasis via FXR, CYP7A1, and multiple bile acid transporters—a potential mechanism for its multi-targeted effects—this hypothesis requires further validation through in vivo and in vitro experiments. Further studies are also required to identify the active components of SNS responsible for its multi-target regulation of the BAs homeostasis axis.

## Conclusion

Our study underscores the pivotal role of BAs homeostasis in LF progression. Aberrant BAs levels and composition are likely critical contributors to LF development. SNS mitigates LF by restoring BAs concentration and composition to near-normal levels, thereby re-establishing BAs homeostasis. This protective effect is attributed to the multi-target regulation of BAs metabolism by SNS, encompassing modulation of BAs-metabolizing enzymes, transporters, gut microbiota, and FXR-mediated negative feedback mechanisms. These findings highlight the therapeutic potential of SNS in maintaining BAs balance and combating LF.

## Supplementary Information

Additional file 1.

## Data Availability

All data included in this article are available from the corresponding author.

## Abbreviations

ALT: Alanine aminotransferase
Asbt: Apical sodium-dependent bile acid transporter
AST: Aspartate aminotransferase
BAs: Bile acids
Bax: Bcl2-Associated X
Bcl-2: B-cell lymphoma-2
Bsep: Bile salt export pump
CA: Cholic acid
CCl_4_: Carbon tetrachloride
CDCA: Chenodeoxycholic acid
CYP27A1: Cytochrome P450 27A1
CYP7A1: Cholesterol 7-alpha hydroxylase
CYP8B1: Cytochrome P450 family 8 subfamily B member 1
DCA: Deoxycholic acid
ERK: Extracellular regulated protein kinases
FXR: Farnesoid X receptor
GCA: Glycocholic acid
GCDCA: Glycochenodeoxycholic acid
HBV: Hepatitis B virus
HCC: Hepatocellular carcinoma
HDCA: Hyodeoxycholic acid
HE: Hematoxylin-eosin
HFC: High-fat, high-cholesterol
HFHS: High-fat, high-sugar diet
HPLC: High-performance liquid chromatography
JNK: C-Jun N-terminal kinase.
LCA: Lithocholic acid
LC–MS/MS: Liquid chromatography-tandem mass spectrometry
LDA: Linear discriminant analysis
LF: Liver fibrosis
MAPK: Mitogen-activated protein kinase
MEK: Mitogen-activated protein kinase kinase
NAFLD: Non-alcoholic fatty liver disease
Ntcp: Sodium taurocholate cotransporting polypeptide
OCA: Obeticholic acid
PBAs: Primary bile acids
PBC: Primary biliary cholangitis
RT-qPCR: Reverse transcription-quantitative PCR
SBAs: Secondary bile acids
S1PR2: Sphingosine-1-phosphate receptor 2
SNS: Sini-San
TBA: Total bile acid
TCA: Taurocholic acid
TCDCA: Taurochenodeoxycholic acid
TDCA: Taurochenodeoxycholic acid
TGR5: Takeda G protein-coupled receptor 5
THDCA: Taurohyodeoxycholic acid
TLCA: Taurolithocholic acid
TUDCA: Tauroursodeoxycholic acid
TUNEL: Terminal deoxynucleotidyl transferase-mediated dUTP-biotin nick end labeling assay
T-βMCA: Tauro-β-muricholic acid
UDCA: Ursodeoxycholic acid
YAP: Yes-associated protein
α-MCA: α-Muricholic acid
α-SMA: Alpha smooth muscle actin
β-MCA: β-Muricholic acid

## References

[1] Moon AM, Singal AG, Tapper EB. Contemporary epidemiology of chronic liver disease and cirrhosis. Clin Gastroenterol Hepatol. 2020;18:2650–66. 31401364 10.1016/j.cgh.2019.07.060 PMC7007353

[2] Roehlen N, Crouchet E, Baumert TF. Liver fibrosis: mechanistic concepts and therapeutic perspectives. Cells. 2020;9:875. 32260126 10.3390/cells9040875 PMC7226751

[3] Winston JA, Theriot CM. Diversification of host bile acids by members of the gut microbiota. Gut microbes. 2020;11:158–71. 31595814 10.1080/19490976.2019.1674124 PMC7053883

[4] Zhang J, Lyu A, Wang C. The molecular insights of bile acid homeostasis in host diseases. Life Sci. 2023;330:121919. 37422071 10.1016/j.lfs.2023.121919

[5] Zhang M, Xiao B, Chen X, Ou B, Wang S. Physical exercise plays a role in rebalancing the bile acids of enterohepatic axis in non-alcoholic fatty liver disease. Acta Physiol (Oxf). 2024;240:e14065. 38037846 10.1111/apha.14065

[6] Chopyk DM, Grakoui A. Contribution of the intestinal microbiome and gut barrier to hepatic disorders. Gastroenterology. 2020;159:849–63. 32569766 10.1053/j.gastro.2020.04.077 PMC7502510

[7] Xiang D, Yang J, Liu L, Yu H, Gong X, Liu D. The regulation of tissue-specific farnesoid X receptor on genes and diseases involved in bile acid homeostasis. Biomed Pharmacother. 2023;168:115606. 37812893 10.1016/j.biopha.2023.115606

[8] Yu S, He J, Zhang Y. Clinical study on 42 cases of hepatic fibrosis caused by chronic hepatitis B with liver stagnation and spleen deficiency syndrome treated by Sini powder combined with Sijunzi decoction and entecavir. Jiangsu J Tradit Chin Med. 2022;54:36–9.

[9] Liu JP, Xing WG. Treating 60 cases of fatty liver with Sini San plus the Weijing decoction. Clin J Chin Med. 2017;9:46–7.

[10] Wu JH, Wu XF, Zhong C, Chen XB. Effect of Ruanjian Sini Powder Combined with Transcatheter Arterial Chemoembolization on T Lymphocyte Subsets,GP-73 and GPC-3Levels in Patients with Primary Hepatocellular Carcinoma. J Guangzhou Univ Tradit Chin Med. 2022;39:19–24.

[11] Jiang M, Huang C, Wu Q, Su Y, Wang X, Xuan Z, et al. Sini san ameliorates CCl4-induced liver fibrosis in mice by inhibiting AKT-mediated hepatocyte apoptosis. J Ethnopharmacol. 2023;303:115965. 36460296 10.1016/j.jep.2022.115965

[12] Wen J, Yang L, Qin F, Zhao L, Xiong Z. An integrative UHPLC-MS/MS untargeted metabonomics combined with quantitative analysis of the therapeutic mechanism of Si-Ni-San. Anal Biochem. 2019;567:128–35. 30367881 10.1016/j.ab.2018.10.023

[13] Wang Y, Li J, Wu L, Qin X, Xie C, Gao X. Saikosaponins regulate bile acid excretion in mice liver and ileum by activating farnesoid X receptor and bile acid transporter. Phytother Res PTR. 2023;37:4572–86. 37318212 10.1002/ptr.7927

[14] Xu X, Hu H, Zeng H, Li B, Yin Q, Jiang Y, et al. Sinisan ameliorates colonic injury induced by water immersion restraint stress by enhancing intestinal barrier function and the gut microbiota structure. Pharm Biol. 2023;61:598–609. 37013944 10.1080/13880209.2023.2191643 PMC10075512

[15] Wang A, Yang X, Lin J, Wang Y, Yang J, Zhang Y, et al. Si-Ni-San alleviates intestinal and liver damage in ulcerative colitis mice by regulating cholesterol metabolism. J Ethnopharmacol. 2025;336:118715. 39179058 10.1016/j.jep.2024.118715

[16] Liu F, Tang X, Mao B, Zhang Q, Zhao J, Cui S, et al. Ethanol extract of licorice alleviates HFD-induced liver fat accumulation in association with modulation of gut microbiota and intestinal metabolites in obesity mice. Nutrients. 2022;14:4180. 36235833 10.3390/nu14194180 PMC9572531

[17] Wu Q, Zhu F, Yao Y, Chen L, Ding Y, Su Y, et al. Sini san regulates intestinal flora and short-chain fatty acids to ameliorate hepatocyte apoptosis and relieve CCl(4)-induced liver fibrosis in mice. Front Pharmacol. 2024;15:1408459. 39281277 10.3389/fphar.2024.1408459 PMC11392872

[18] Zhou J, Huang N, Guo Y, Cui S, Ge C, He Q, et al. Combined obeticholic acid and apoptosis inhibitor treatment alleviates liver fibrosis. Acta Pharm Sin B. 2019;9:526–36. 31193776 10.1016/j.apsb.2018.11.004 PMC6542786

[19] Su Y, Zhou Q, Wu Q, Ding Y, Jiang M, Zhang X, et al. Infection-associated bile acid disturbance contributes to macrophage activation in patients with cirrhosis. Mol Med Rep. 2024;30:150. 38963032 10.3892/mmr.2024.13274 PMC11234163

[20] 2020. Pharmacopoeia of the People’s Republic of China. In: Beijing: China Medical Science Press; 2020.

[21] Li Y, Bian T, Cao R, Niu J, Si X, Yan X. Establishment of quality standard of HPLC for Si-Ni-San powder. Chin J Clin Pharmacol. 2019;35:65–8.

[22] Yang M, Qi X, Li N, Kaifi JT, Chen S, Wheeler AA, et al. Western diet contributes to the pathogenesis of non-alcoholic steatohepatitis in male mice via remodeling gut microbiota and increasing production of 2-oleoylglycerol. Nat Commun. 2023;14:228. 36646715 10.1038/s41467-023-35861-1 PMC9842745

[23] Ichimura-Shimizu M, Watanabe S, Kashirajima Y, Nagatomo A, Wada H, Tsuneyama K, et al. Dietary cholic acid exacerbates liver fibrosis in NASH model of Sprague-Dawley rats fed a high-fat and high-cholesterol diet. Int J Mol Sci. 2022;23:9268. 36012527 10.3390/ijms23169268 PMC9409005

[24] Duboc H, Rajca S, Rainteau D, Benarous D, Maubert MA, Quervain E, et al. Connecting dysbiosis, bile-acid dysmetabolism and gut inflammation in inflammatory bowel diseases. Gut. 2013;62:531–9. 22993202 10.1136/gutjnl-2012-302578

[25] Mukherjee A, Lordan C, Ross RP, Cotter PD. Gut microbes from the phylogenetically diverse genus *Eubacterium* and their various contributions to gut health. Gut microbes. 2020;12:1802866. 32835590 10.1080/19490976.2020.1802866 PMC7524325

[26] Yang J, Tang X, Liang Z, Chen M, Sun L. Taurocholic acid promotes hepatic stellate cell activation via S1PR2/p38 MAPK/YAP signaling under cholestatic conditions. Clin Mol Hepatol. 2023;29:465–81. 36800698 10.3350/cmh.2022.0327 PMC10121313

[27] Hohenester S, Kanitz V, Kremer AE, Paulusma CC, Wimmer R, Kuehn H, et al. Glycochenodeoxycholate promotes liver fibrosis in mice with hepatocellular cholestasis. Cells. 2020;9:281. 31979271 10.3390/cells9020281 PMC7072501

[28] Hasan MN, Chen J, Wang H, Du Y, Clayton YD, Gu L, et al. Glycine-β-muricholic acid improves liver fibrosis and gut barrier function by reducing bile acid pool size and hydrophobicity in male Cyp2c70 knockout mice. Cells. 2023;12:1371. 37408204 10.3390/cells12101371 PMC10216635

[29] Nevens F, Andreone P, Mazzella G, Strasser SI, Bowlus C, Invernizzi P, et al. A placebo-controlled trial of Obeticholic acid in primary biliary cholangitis. N Engl J Med. 2016;375:631–43. 27532829 10.1056/NEJMoa1509840

[30] Cai H, Zhang J, Liu C, Le TN, Lu Y, Feng F, et al. High-fat diet-induced decreased circulating bile acids contribute to obesity associated with gut microbiota in mice. Foods. 2024;13:699. 38472812 10.3390/foods13050699 PMC10931208

[31] Collins SL, Stine JG, Bisanz JE, Okafor CD, Patterson AD. Bile acids and the gut microbiota: metabolic interactions and impacts on disease. Nat Rev Microbiol. 2023;21:236–47. 36253479 10.1038/s41579-022-00805-x PMC12536349

[32] Zhang YL, Li ZJ, Gou HZ, Song XJ, Zhang L. The gut microbiota-bile acid axis: a potential therapeutic target for liver fibrosis. Front Cell Infect Microbiol. 2022;12:945368. 36189347 10.3389/fcimb.2022.945368 PMC9519863

[33] Adams LA, Wang Z, Liddle C, Melton PE, Ariff A, Chandraratna H, et al. Bile acids associate with specific gut microbiota, low-level alcohol consumption and liver fibrosis in patients with non-alcoholic fatty liver disease. Liver Int. 2020;40:1356–65. 32243703 10.1111/liv.14453

[34] Wang X, Chen L, Wang H, Cai W, Xie Q. Modulation of bile acid profile by gut microbiota in chronic hepatitis B. J Cell Mol Med. 2020;24:2573–81. 31925905 10.1111/jcmm.14951 PMC7028859

[35] Xie G, Jiang R, Wang X, Liu P, Zhao A, Wu Y, et al. Conjugated secondary 12α-hydroxylated bile acids promote liver fibrogenesis. EBioMedicine. 2021;66:103290. 33752128 10.1016/j.ebiom.2021.103290 PMC8010625

[36] Yang M, Gu Y, Li L, Liu T, Song X, Sun Y, et al. Bile acid-gut microbiota axis in inflammatory bowel disease: from bench to bedside. Nutrients. 2021;13:3143. 34579027 10.3390/nu13093143 PMC8467364

[37] Fu K, Ma C, Wang C, Zhou H, Gong L, Zhang Y, et al. Forsythiaside A alleviated carbon tetrachloride-induced liver fibrosis by modulating gut microbiota composition to increase short-chain fatty acids and restoring bile acids metabolism disorder. Biomedicine & pharmacotherapy = Biomedecine & pharmacotherapie. 2022;151:113185. 35623173 10.1016/j.biopha.2022.113185

[38] Velázquez KT, Enos RT, Bader JE, Sougiannis AT, Carson MS, Chatzistamou I, et al. Prolonged high-fat-diet feeding promotes non-alcoholic fatty liver disease and alters gut microbiota in mice. World J Hepatol. 2019;11:619–37. 31528245 10.4254/wjh.v11.i8.619 PMC6717713

[39] Ju T, Kong JY, Stothard P, Willing BP. Defining the role of *Parasutterella*, a previously uncharacterized member of the core gut microbiota. ISME J. 2019;13:1520–34. 30742017 10.1038/s41396-019-0364-5 PMC6776049

[40] Li M, Wang Q, Niu M, Yang H, Zhao S. Protective effects of insoluble dietary fiber from cereal bran against DSS-induced chronic colitis in mice: from inflammatory responses, oxidative stress, intestinal barrier, and gut microbiota. Int J Biol Macromol. 2024;283:137846. 39566792 10.1016/j.ijbiomac.2024.137846

[41] Rodriguez-Diaz C, Taminiau B, García-García A, Cueto A, Robles-Díaz M, Ortega-Alonso A, et al. Microbiota diversity in nonalcoholic fatty liver disease and in drug-induced liver injury. Pharmacol Res. 2022;182:106348. 35817360 10.1016/j.phrs.2022.106348

[42] Chiang JYL, Ferrell JM. Discovery of farnesoid X receptor and its role in bile acid metabolism. Mol Cell Endocrinol. 2022;548:111618. 35283218 10.1016/j.mce.2022.111618 PMC9038687

[43] Song L, Hou Y, Xu D, Dai X, Luo J, Liu Y, et al. Hepatic FXR-FGF4 is required for bile acid homeostasis via an FGFR4-LRH-1 signal node under cholestatic stress. Cell Metab. 2025;37:104-20.e9. 39393353 10.1016/j.cmet.2024.09.008

[44] Chen J, Zhang L, Chen Y, Yan Y, Lu C. Alpha-tocopheryl quinone attenuates liver fibrosis through enriching *Christensenella minuta* and modulating bile acids metabolism via gut-liver axis. Phytomedicine. 2025;146:157108. 40768805 10.1016/j.phymed.2025.157108

[45] Lu JL, Yu CX, Song LJ. Programmed cell death in hepatic fibrosis: current and perspectives. Cell Death Discov. 2023;9:449. 38086792 10.1038/s41420-023-01749-8 PMC10716404

[46] Liu T, Lei C, Huang Q, Song W, Li C, Sun N, et al. Hesperidin and fecal microbiota transplantation modulate the composition of the gut microbiota and reduce obesity in high fat diet mice. Diabetes Metab Syndr Obes Targets Ther. 2024;17:3643–56. 10.2147/DMSO.S474034 PMC11468570 39398388

[47] Yu JB, Zhao ZX, Peng R, Pan LB, Fu J, Ma SR, et al. Gut microbiota-based pharmacokinetics and the antidepressant mechanism of Paeoniflorin. Front Pharmacol. 2019;10:268. 30949054 10.3389/fphar.2019.00268 PMC6435784

[48] Fan Q, Guan X, Hou Y, Liu Y, Wei W, Cai X, et al. Paeoniflorin modulates gut microbial production of indole-3-lactate and epithelial autophagy to alleviate colitis in mice. Phytomedicine. 2020;79:153345. 33002829 10.1016/j.phymed.2020.153345

[49] Liu N, Meng Z, Lou G, Zhou W, Wang X, Zhang Y, et al. Hepatocarcinogenesis in FXR-/- mice mimics human HCC progression that operates through HNF1α regulation of FXR expression. Mol Endocrinol (Baltimore, Md). 2012;26:775–85. 10.1210/me.2011-1383 PMC3355555 22474109

[50] Yang F, Huang X, Yi T, Yen Y, Moore DD, Huang W. Spontaneous development of liver tumors in the absence of the bile acid receptor farnesoid X receptor. Cancer Res. 2007;67:863–7. 17283114 10.1158/0008-5472.CAN-06-1078

