# Supplementary material for: Regulatory effects of Sini-San on bile acid homeostasis in the enterohepatic circulation of mice with liver fibrosis
Source: Chin Med. 2025 Nov 11;20:186. doi: 10.1186/s13020-025-01252-5 (PMC12604316; doi:10.1186/s13020-025-01252-5)
Supplement: Supplementary file 1 — Additional file 1. [file 13020_2025_1252_MOESM1_ESM.docx]

**Supplemental Materials**

**Figures**


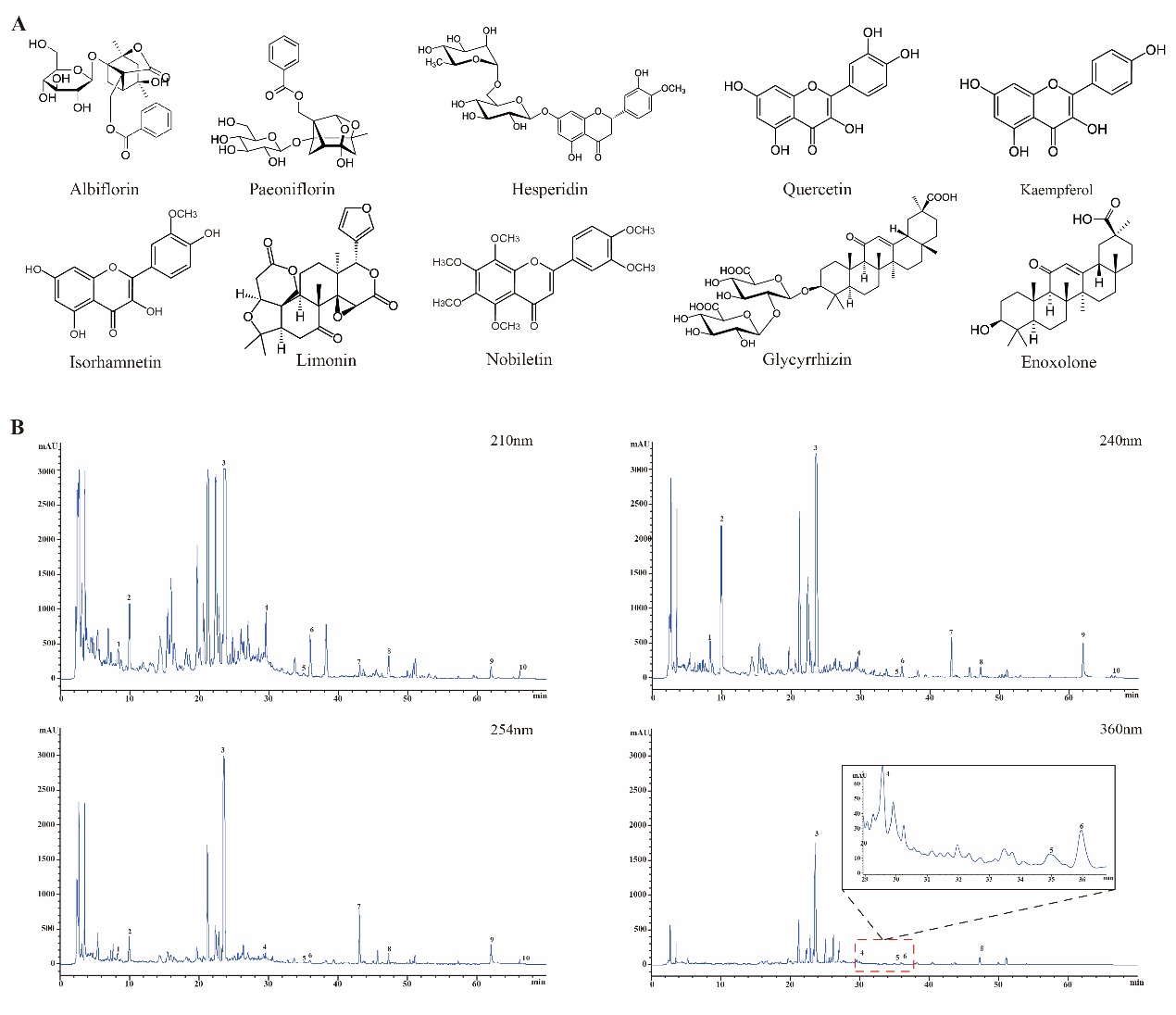


**Fig.S1 Compositional analysis of SNS.** (A) Structural formulas of 10 standards. (B) HPLC chromatograms of test samples at 210 nm, 240 nm, 250 nm and 360 nm. 1: Albiflorin 2: Paeoniflorin 3: Hesperidin 4: Quercetin 5: Kaempferol 6: Isorhamnetin 7: Limonin 8: Nobiletin 9: Glycyrrhizin 10: Enoxolone


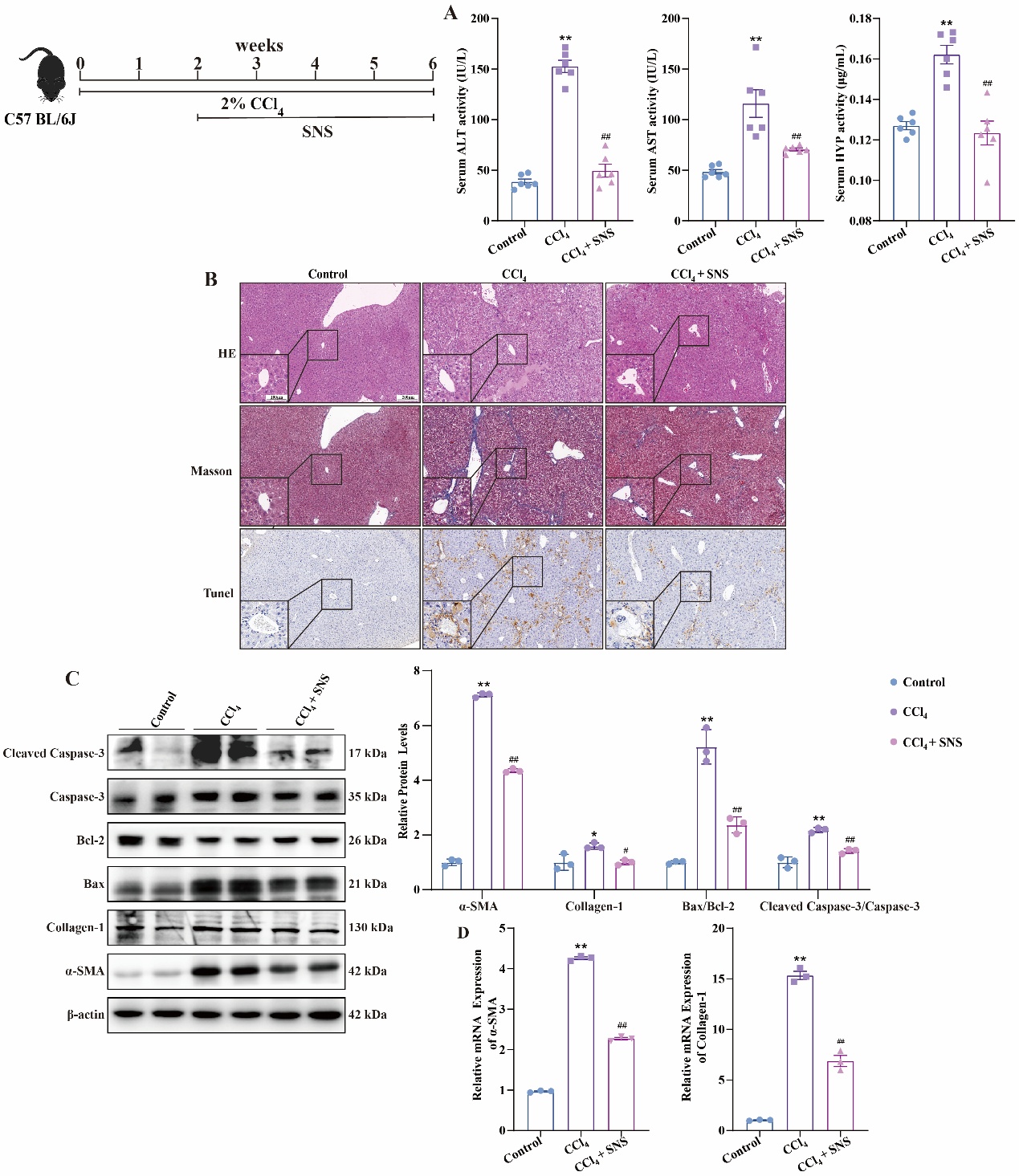


**Fig.S2 Effects of SNS on liver function, liver fibrosis and apoptosis in CCl_4_-induced liver fibrosis mice.** (A) Serum levels of ALT, AST and HYP (n = 6). (B) HE, Masson, and TUNEL staining of liver tissue (n = 3). (C) Western blotting analysis and relative quantification of α-SMA, Collagen-1, Bax, Bcl-2, cleaved Caspase-3 and Caspase-3 (n = 3). (D) The mRNA expression of α-SMA and Collagen-1 was detected by RT-qPCR. (n = 3). **p* < 0.05, ***p* < 0.01 VS Control group; *^#^p* < 0.05, *^##^p* < 0.01 VS CCl_4_ group.


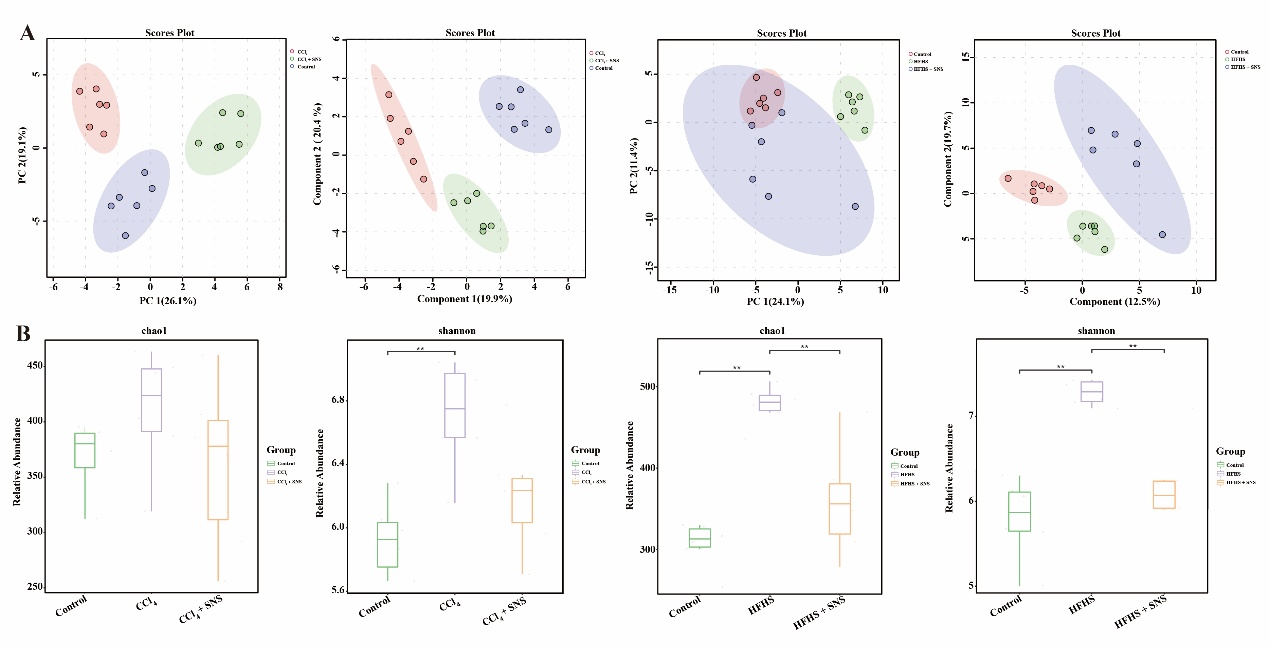


**Fig.S3 α and β diversity analysis of intestinal microbiota in the two models.** (A) Principal component analysis (PCA) and partial least squares discriminant analysis (PLS-DA) for β-diversity. (B) α-diversity box plot showing the abundance and diversity of intestinal flora. Each symbol represents one mouse. Data pooled from one independent experiment, n = 6. **p < 0.05.*


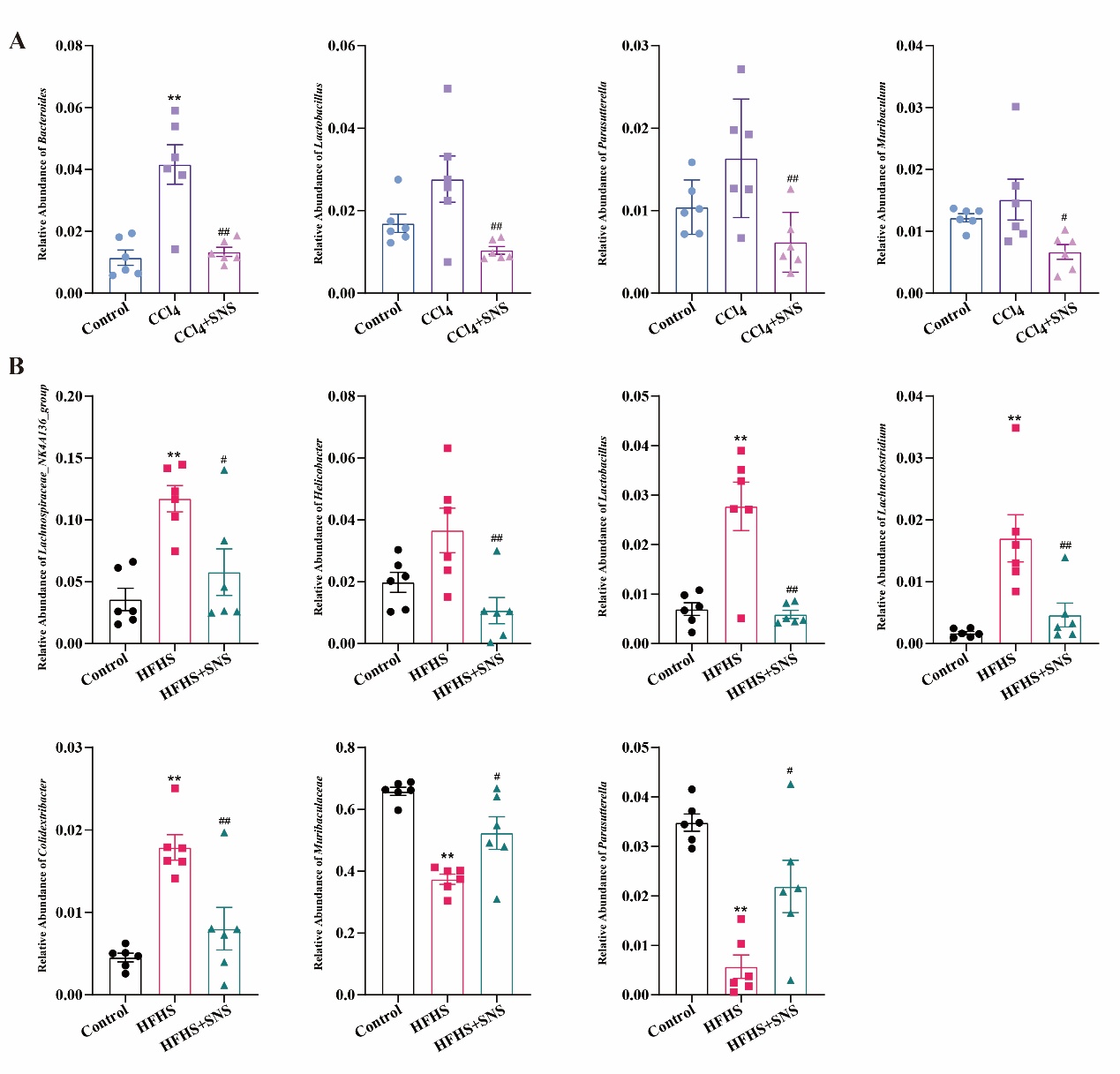


**Fig.S4 The effect of SNS on the content of BAs-associated flora. (**A) Relative abundance of intestinal microbiota in the CCl_4_ model. (B) Relative abundance of intestinal microbiota in the HFHS model. Each symbol represents one mouse. Data pooled from one independent experiment, n = 6. **p < 0.05.*


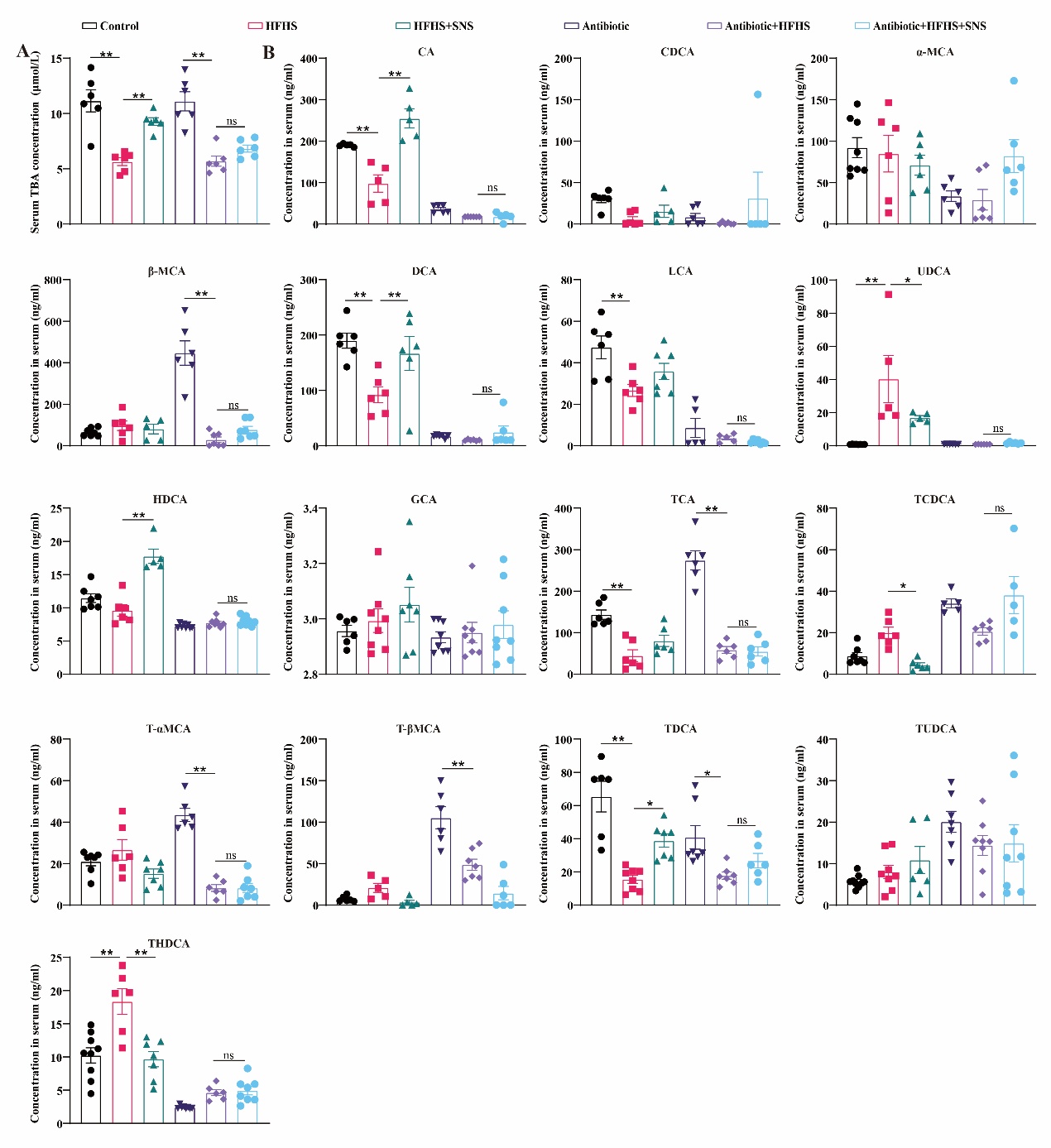


**Fig.S5 Impact of SNS on BAs metabolism in CCl_4_ model in pseudo-sterile mice.** (A) Serum TBA levels in the mice (n = 6). (B) Serum BAs profile in the model (n = 6). **p < 0.05.*

**
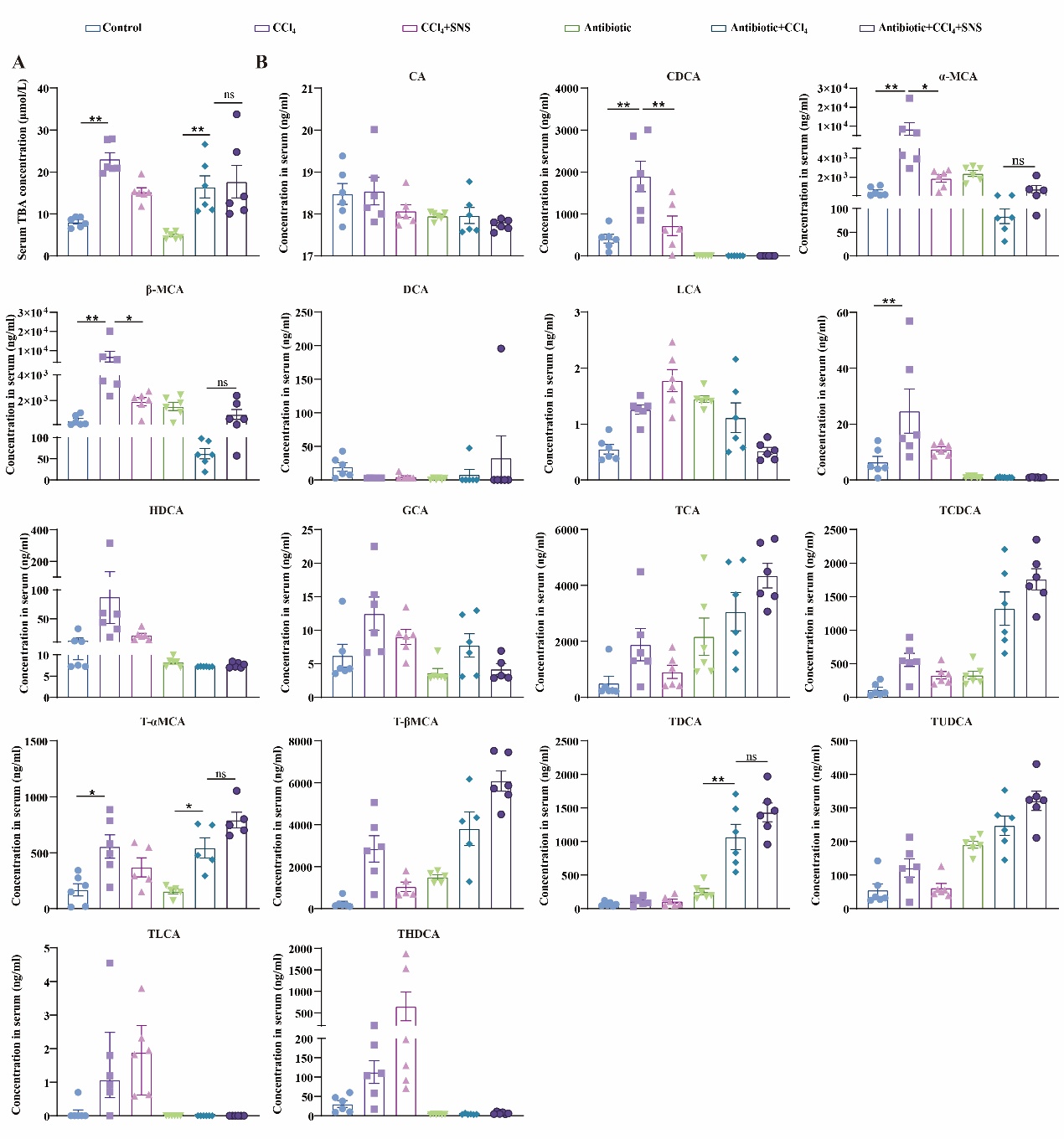
**

**Fig.S6 Impact of SNS on BAs metabolism in HFHS model in pseudo-sterile mice.** (A) Serum TBA levels in the mice (n = 6). (B) Serum BAs profile in the model (n = 6). **p < 0.05.*

**Tables**

**Table S1 Reagents and Materials**

| Name | Manufacturer | Catalog Number | Dilution Ratio |
| --- | --- | --- | --- |
| β-actin | Proteintech | 66009-1-Ig | 1:20000 |
| Cyp7a1 | Proteintech | 18054-1-AP | 1:1000 |
| Bax | Proteintech | 50599-2-Ig | 1:1000 |
| Caspase-3 | Proteintech | 19677-1-AP | 1:1000 |
| Collagen-I | Boaosen | bs-10423R | 1:1000 |
| α-SMA | Huaan | ET1607-53 | 1:1000 |
| Cyp27a1 | Huaan | ET7109-05 | 1:1000 |
| FXR | Huaan | ER1914-12 | 1:1000 |
| Bcl-2 | Zhengneng | 381702 | 1:1000 |
| HRP-conjugated Goat Anti-Rabbit IgG(H+L) | Proteintech | SA00001-2 | 1:10000 |
| HRP-conjugated Goat Anti-Mouse IgG(H+L) | Proteintech | SA00001-1 | 1:10000 |

**Table S2 Primer sequences of real-time polymerase chain reaction**

|  | Forward (5’to3’) | Reverse (5’to3’) |
| --- | --- | --- |
| Mouse *Gapdh* | TTGAGGTCAATGAAGGGGTC | TCGTCCCGTAGACAAAATGG |
| Mouse *α-SMA* | GGCACCACTGAACCCTAAGG | ACAATACCAGTTGTACGTCCAGA |
| Mouse *Collagen-1* | TAAGGGTCCCCAATGGTGAGA | GGGTCCCTCGACTCCTACAT |
| Mouse *FXR* | GGCAGAATCTGGATTTGGAATCG | GCCCAGGTTGGAATAGTAAGACG |
| Mouse *Cyp7a1* | GAACCTCCTTTGGACAACGGG | GGAGTTTGTGATGAAGTGGACAT |
| Mouse *Cyp27a1* | GCACAGGAGAGTACGGAGG | CGGGCAAGTGCAGCACATA |
| Mouse *BSEP* | TCTGACTCAGTGATTCTTCGCA | CCCATAAACATCAGCCAGTTGT |
| Mouse *NTCP* | CAAACCTCAGAAGGACCAAACA | GTAGGAGGATTATTCCCGTTGTG |
| Mouse *ASBT* | TTGCACAGCACAAGCAGTGA | TGCATTGAAGTTGCTCTCAGGT |
| Mouse *OATP* | CAATTCGGTATCCCCACGTCT | CTGCACATCCTACACCAATCAT |
